# HIF-1α/Wnt signaling-dependent control of gene transcription regulates neuronal differentiation of glioblastoma stem cells

**DOI:** 10.7150/thno.35882

**Published:** 2019-07-09

**Authors:** Daniele Boso, Elena Rampazzo, Carlo Zanon, Silvia Bresolin, Francesca Maule, Elena Porcù, Alice Cani, Alessandro Della Puppa, Luca Trentin, Giuseppe Basso, Luca Persano

**Affiliations:** 1 Department of Woman and Children's Health, University of Padova, Padova, Italy.; 2 Istituto di Ricerca Pediatrica - Città della Speranza - IRP, Padova, Italy.; 3 Neurosurgery Unit, Padova University Hospital, Padova, Italy.

**Keywords:** Cancer stem cells, glioblastoma, HIF-1α, neuronal differentiation.

## Abstract

HIF-1α has been suggested to interplay with Wnt signaling components in order to activate a neuronal differentiation process in both normal brain and glioblastoma (GBM). Based on these data, we explored the molecular mechanisms underlying the observed capability of GBM cells to acquire a neuronal phenotype upon Wnt signaling stimulation and how the microenvironment, particularly hypoxia, modulates this process.

**Methods**: here, the employment of ChIP-seq techniques together with co-immunoprecipitation approaches allowed to reconstruct the molecular interactions responsible for activating specific pro-differentiating transcriptional programs in GBM cells. Moreover, gene silencing/over-expression approaches coupled with the functional analysis of cell phenotype were applied to confirm ChIP-driven hypotheses. Finally, we combined the use of publicly available gene expression datasets with protein expression data by immunohistochemistry to test the clinical relevance of obtained results.

**Results**: our data clearly suggest that HIF-1α is recruited by the β-catenin/TCF1 complex to foster neuronal differentiation gene transcription in hypoxic GBM cells. Conversely, at higher oxygen levels, the increased expression of TCF4 exerts a transcriptional inhibitory function on the same genomic regions, thus counteracting differentiation. Moreover, we demonstrate the existence of a positive correlation between the expression levels of HIF-1α, TCF1 and neuronal phenotype in GBM tumors, accompanied by the over-expression of several Wnt signaling components, finally affecting patient prognosis.

**Conclusion**: we unveiled a peculiar mechanism by which TCF1 and HIF-1α can induce a reminiscent neuronal differentiation of hypoxic GBM cells, which is hampered, in normoxia, by high levels of TCF4, thus not only *de facto* controlling the balance between differentiation and stemness, but also impacting on intra-tumoral heterogeneity and eventually patient outcome.

## Introduction

Hypoxia and its major sensor Hypoxia Inducible Factor (HIF)-1α are fundamental hallmarks of the niches in which stem cells, particularly neural stem cells (NSCs), reside, here sustaining their self-renewal and multipotent phenotype 1, 2. This knowledge becomes particularly relevant when transferred to a cancer setting in which a dramatic HIF-1α stabilization often occurs as a result of uncontrolled cell growth, aberrant vascularization or either a glycolytic metabolic shift 3-6. In this context, glioblastoma (GBM) is the most aggressive and deadly brain tumor, histologically characterized by extensive areas of necrosis and described to be highly hypoxic 7, 8. In GBM, hypoxia plays a crucial role in sustaining cancer (stem) cell growth and their intrinsic radio- and chemo-resistance 9-13.

Among the several pathways modulated by hypoxia, the Wnt/β-catenin/TCF signaling is one of the most important, whose implication in tissue development and progression of several human cancers, particularly colorectal tumors, has been extensively investigated 14-16. The central player of canonical Wnt signaling is β-catenin. In particular, the interaction of Wnt ligands with specific receptors promotes the translocation of β-catenin into the nucleus, where it binds to members of the TCF/LEF family of co-factors in order to efficiently transactivate their targets genes 17. Similarly to other high-mobility group (HMG) box-containing proteins, TCF/LEF factors possess a limited transcriptional activity by their own, thus requiring the binding of several co-factors and the recruitment of peculiar chromatin modifiers to activate/suppress target gene transcription 18. Interestingly, we and others previously suggested that hypoxia co-operates with Wnt signaling by sustaining the over-expression of TCF1 and LEF1 in both normal brain 15 and GBM 19. Moreover, we reported that Wnt signaling activation under hypoxic conditions is enough to induce a dramatic neuronal differentiation of GBM stem-like cells 19. Based on this knowledge, we hypothesized that in GBM, hypoxia-induced HIF-1α stabilization could act as a molecular tuner of the transcriptional response to Wnt signaling activation. In particular, our previous data suggested that hypoxia may modulate the balance of multiple Wnt pathway co-factors eventually involved in the transcriptional control of cancer stem cell (CSC) survival and/or differentiation. Here, we show that Wnt pathway activation induces a switch from a stem-like phenotype towards neurons and triggers, exclusively under hypoxia, a TCF1/HIF-1α-dependent activation of genes involved in promoting neuronal differentiation of GBM cells. Moreover, we demonstrate that this process is impaired under normoxic conditions (20% O_2_) due to a strong up-regulation of high molecular weight (hMW) TCF4 isoforms that, in turn, act as transcriptional inhibitors of this process. In conclusion, we unveil a tightly regulated mechanism by which HIF-1α controls the balance of Wnt signaling co-factors and how their molecular interplay regulates the transcriptional events responsible for the phenotypic shift of GBM stem cells toward a reminiscent neuronal differentiation. This knowledge might represent a future potential strategy to therapeutically weaken GBM aggressiveness.

## Materials and Methods

### Neurosurgical sample collection, isolation and gas-controlled expansion of GBM cells

Written informed consent for the donation of adult tumor brain tissues was obtained from patients before surgery under the auspices of the protocol for the acquisition of human brain tissues obtained from the Ethical Committee of the Padova University Hospital. All tissues were acquired following the tenets of the Declaration of Helsinki. A differential sampling of tumor biopsies from either the GBM core or the more peripheral regions of the mass has been achieved through a T1-weighted MRI-based intra-operative neuro-navigation and image-guided collection of pre-identified GBM biopsies 11, 20. General clinical features of patients from which GBM primary cultures used in this study have been derived are listed in Table S1. Primary GBM cells were isolated and maintained in culture as described previously 11, 20. Briefly, tumor biopsies were subjected to mechanical and enzymatic dissociation and the resulting cell suspension was cultured on fibronectin-coated dishes in DMEM/F12 medium supplemented with BIT9500 (Stemcell Technologies Inc., Vancouver, Canada), 20ng/ml basic Fibroblast Growth Factor (bFGF; Sigma-Aldrich, St. Louis, MO) and 20ng/ml Epidermal Growth Factor (EGF; R&D Systems, Minneapolis, MN). Immediately after isolation, but also during standard culturing conditions GBM cells were maintained in an atmosphere of 2% oxygen, 5% carbon dioxide and balanced nitrogen in an INVIVO2 300 (Ruskinn Technology Ltd, Bridgend, UK) or, alternatively, a H35 (Don Whitley Scientific Ltd, Shipley, UK) hypoxystations in order to select the proliferating and relatively “stem-like” subpopulation of cells and prevent their differentiation. Cells were eventually exposed to normoxic (20% O_2_) conditions, depending on specific experimental needs. Wnt signaling activation has been achieved by treating GBM cells with recombinant Wnt3a (30ng/ml; Peprotech, Rocky Hill, NJ) until specific time-points. In some experiments Wnt3a pre-treated or control cells have been exposed to scalar doses of temozolomide (TMZ) and viability measured by MTT assay.

In a small subset of experiments, fetal neuron LUHMES cells (ATCC^®^ CRL-2927™) have been cultured under standard (DMEM:F12 medium with 1%N2 supplement (Invitrogen, Carlsbad, CA) and 40ng/ml bFGF) or differentiating conditions, which consist in 10 days of culture after the addition of GDNF (Miltenyi Biotec, Bergisch Gladbach, Germany), tetracycline and db-cAMP (both from Sigma Aldrich, St. Louis, MO) in the culture medium, and then total RNA extracted with standard procedures.

### Immunofluorescence

GBM cells were cultured on 4-well chamber slides (BD Bioscience, San Jose, CA), treated depending on the experimental plan, fixed in cold 4% formaldehyde and stored at +4°C prior to analysis. After incubation with primary antibodies including anti-TCF7 (1:100, Sigma-Aldrich, St. Louis, MO, #WH0006932M1), anti-TCF7L2 (1:50, Sigma-Aldrich, #SAB1409729), anti-Nestin (1:200, Millipore, Burlington, MA, #MAB5326), anti-β-III tubulin (1:1000, Covance, Princeton, NJ, #MMS-435P), cells were washed and incubated with species-specific secondary antibodies conjugated to Alexa dyes (1:2000, Thermo Fisher Scientific, Waltham, MA). Cells were counterstained with DAPI (1:10000; Sigma-Aldrich, #D9542). Staining was visualized by epifluorescence with a ViCo microscope (Vico, Nikon, Melville, NY).

### Neurosphere forming and limiting dilution assays

Self-renewal capacity of primary GBM cells was assessed as follows: the first day 2×10^5^ cells/well were seeded onto fibronectin-coated six-well plates. At day one, cells were treated with Wnt3a (30ng/ml) under hypoxic or normoxic conditions (untreated cells were used as control). At day 6, cells were trypsinized and seeded onto uncoated 6-well plates at 10^4^ cells/well. After a week, neurospheres were counted and dissociated with TrypLE™ Express (Thermo Fischer Scientific Inc., Waltham, MA). After neurosphere disaggregation, 10^4^ GBM cells were re-seeded onto uncoated 6-well plates for obtaining a second and then a third generation of neurospheres.

To assess the GBM initiating cell frequency we seeded GBM cells in 6 well plates under hypoxic and normoxic conditions. Then, we treated them with Wnt3a (30ng/ml) for 3 days and, at day 6, we re-plated serial dilutions of cells ranging from 0 to 500 cells/well in 96 well plates. Cells were cultured under low or high oxygen levels for two additional weeks and then the proportion of wells in which sphere formation has not been observed was then calculated.

### Western blot and immunoprecipitation

Equal amounts of proteins extracted from primary GBM cells (10µg) were resolved by SDS-PAGE gels and transferred to polyvinylidene difluoride (PVDF) Immobilon-p membrane (Merk-Millipore, Darmstadt, Germany). Membranes were blocked with I-block™ (Thermo Fisher Scientific, Waltham, MA) for at least 1 hour at room temperature and then incubated overnight at +4°C under constant shaking with these primary antibodies: anti-TCF1 (1:500, Cell Signaling Technologies, Danvers, MA, #2203), anti-TCF7 (TCF1; 1:100, Sigma-Aldrich, St. Louis, MO, #WH0006932M1), anti-TCF7L2 (TCF4; 1:50, Sigma-Aldrich, #SAB1409729), anti-TCF4 (1:500, Cell Signaling Technologies, #2569), anti-HIF-1α (1:100, BD Biosciences, San Jose, CA, #610958), anti-β-catenin (1:1000, Abcam, Cambridge, UK, #ab2365), anti-β-actin (1:25000, Sigma-Aldrich, St. Louis, MO, #A2228). Membranes were next incubated with peroxidase-conjugated secondary antibodies, visualized using ECL Select and exposed to Amersham Hyperfilm ECL (both from GE Healthcare, Little Chalfont, UK).

For immunoprecipitation (IP) experiments GBM cells were treated or not with Wnt3a (30ng/ml) for 48 hours in hypoxic or normoxic conditions. Cells were then solubilized in lysis buffer (MgCl_2_ 1M, KCl 1M, EDTA 0,5M, TRIS-HCl pH 7.5, Chapso 1%). A small quantity of cell lysate was collected prior to binding with antibodies and labeled as Input. Then, an equal amount of each lysate was incubated with anti-β-catenin (4µg, Abcam, Cambridge, UK, #ab2365) or anti-HIF-1α (4µg, Abcam, #ab2185), followed by incubation with protein A/G-Microbeads (µMACS^TM^ MultiMACS Protein A/G kit, Miltenyi Biotec, Bergisch Gladbach, Germany). Irrelevant IgGs were used as negative control. Immune complexes were analyzed by Western blot as described above.

### Chromatin Immunoprecipitation-Sequencing (ChIP-seq) and data analysis

For ChIP-seq experiments we immunoprecipitated HIF-1α, TCF1 and TCF4 in control or either Wnt3a-treated (30ng/ml) GBM cells under hypoxic/normoxic conditions. ChIP-seq experiments were performed as previously described 21, 22 with limited modifications. In particular, primary GBM cells were cross-linked and washed. Then, cells were lysed in Lysis buffer 1 (50mM HEPES-KOH, pH 7.5; 140mM NaCl; 1mM EDTA; 10% glycerol; 0.5% NP-40; 0.25% Triton X-100; protease inhibitors) and, after centrifugation, resuspended in Lysis buffer 2 (10mM Tris-HCl, pH 8; 200mM NaCl; 1mM EDTA; 0.5mM EGTA; protease inhibitors). Cells were pelleted and resuspended in Sonication buffer (10mM Tris-HCl, pH 8; 100mM NaCl; 1mM EDTA: 0.5mM EGTA; 0.1% Na-Deoxycholate; 0.05% N-lauroylsarcosine; protease inhibitors) and sonicated in a Bioruptor sonicator (Diagenode, Denville, NJ). Cell lysates were added to protein G beads (Invitrogen, Carlsbad, CA), previously resuspended in 250μl of PBS, 0.5% BSA and 5μg of specific antibody, and incubated overnight at 4°C. A small quantity of cell lysate prior to bead addition was stored as Input. Cross-linking was reversed and DNA extracted by a standard Phenol/Chloroform protocol. For library preparation, all samples were prepared by using the Illumina/Solexa Genomic DNA kit (Illumina, San Diego, CA) according to manufacturer's instructions.

ChIP-seq datasets were aligned using Bowtie2 (version 2.1.0) 23 to the human genome (build hg19) with parameters -k 1 -m 1 -n 2. We used the MACS2 (ver. 2.0.9) 24 to find peaks and identifying regions of signal enrichment over the input DNA control, with the parameters --no-model --keep-dup=1, 'mfold' was set to 5 and 10000, 'q-value' to 0.05 and 'p-value' to 0.0005. The heatmaps in Figure 2A were generated using the 'heatmap.plus' function of the 'heatmap.plus.package' of R statistical software. Transcription factors binding motifs analysis and the related graphical logos were performed using Homer2 software (v 4.9) on the output of MACS2. Known motifs were identified by means of the 'findMotifsGenome' command, with the 'size' parameter set to 750 with the 'length' parameter ranging between 6 and 15.

### Gene expression profiling and data analysis

Frozen GBM biopsies sampled according to the three-layer concentric model (Table S2) 11, 20 were homogenized and total RNA extracted by standard procedures. For microarray experiments, *in vitro* transcription, hybridization and biotin labeling of RNA were performed according to GeneChip™ WT Pico Kit protocol and Clariom™ S human gene platform (Thermofisher, Waltham, MA). Microarray data (CEL files) were generated using default Affymetrix microarray analysis parameters (Command Console Suite Software by Affymetrix). CEL files were normalized using the robust multiarray averaging expression measure of Affy-R package (www.bioconductor.org). Differentially expressed genes between core and periphery-derived GBM samples (n=4) were identified using Significance Analysis of Microarray (SAM) algorithm coded in the samr R package 25. In SAM, we estimated the percentage of false positive predictions (i.e., False Discovery Rate, FDR) with 100 permutations. Genes with an FDR<0.05 were considered significant. Expression data have been deposited into the Gene Expression Omnibus (GEO) database under Series Accession Number GSE113512 and are accessible without restrictions.

Hierarchical clustering analyses were generated by R software (www.R-project.org) using Euclidean distance as a distance measure between genes and Ward.2 method for clustering probe sets. Gene Set Enrichment Analysis (GSEA) was performed using GSEAv2.0 with probe sets ranked by signal-to-noise ratio and statistical significance determined by 1000 permutations 26. Gene set permutations (<7 replicates in each class) were used to enable direct comparisons between GBM core and periphery. For GSEA an FDR cutoff <0.25 and p-value<0.05 were used. MgSigDataBase derived from c2 curated dataset were selected to obtain the gene set enrichments.

Unsupervised hierarchical clustering analyses in Figure S3B and Figure S9F were generated as described above by using gene signatures retrieved from the intersection of HIF-1α/TCF1 consensus (80bp sequence) with genes retrieved from TCF4 IP in normoxia. These gene lists (91 genes in Figure S3B and Table S3; 60 upregulated out of 84 genes in Figure S9F and Table S4) have been applied to a public cohort of glioma and normal brain samples (GSE4290 dataset, 27) or to the expression matrix of core and peripheral GBM tissues generated in our laboratory (GSE113512). Levelplots in Figure 2E and 2F were generated by mediating the expression levels of the top 20 down-regulated genes in glioma of different grade compared to normal samples (GSE4290 dataset) 27 or in hypoxic/normoxic Wnt3a-treated GBM cells, respectively, by using the Morpheus tool (https://software.broadinstitute.org/morpheus/).

For each Wnt component gene (N=44, “Wnt genes”) belonging to the Wnt signaling pathway we calculated a z-score using the TCGA GBM tumor dataset 28 to look for evidence of GBM patients with any “WNT gene” highly upregulated in the array. We determined up-regulated genes>7 and z-score>1.5 as cut-off which significantly divide GBM patients for survival by applying a multivariate Cox analysis (Wald test; p<0.0001).

### Reverse transcription and real-time (RT) PCR

RNA was extracted from GBM cells using TRIzol reagent (Thermo Fisher Scientific, Waltham, MA) according to manufacturer's instructions and 1-2µg of total RNA reverse-transcribed using SuperScript™ III First-Strand Synthesis System (Thermo Fisher Scientific, Waltham, MA). Quantitative RT-PCR reactions were run in triplicate using Platinum SYBR Green Q-PCR Super Mix (Thermo Fisher Scientific, Waltham, MA). Fluorescent emission was recorded in real-time (Sequence Detection System 7900HT, Applied Biosystems, Foster City, CA). The specificity of primers was confirmed for every PCR run by dissociation curve analysis. Primers used are listed in Supplementary Table S5 and specificity confirmed by Human BLAT Search (http://genome.ucsc.edu). Relative RNA quantities were normalized to GUSB expression according to the ΔΔCt Method.

### Transfection of primary GBM cells

To achieve a suitable gene silencing, GBM cells were transfected with 200pmol of small interfering RNAs (Silencer® Select Custom Designed siRNAs, Thermo Fisher Scientific, Waltham, MA) against TCF7 (TCF1; sense: 5'-AUGCUAGGUUCUGGUGUACtt-3', antisense: 5'-GUACACCAGAACCUAGCAUca-3') and TCF7L2 (TCF4; sense: 5'-CACGCCUCUUAUCACGUACtt-3', antisense: 5'-GUACGUGAUAAGAGGCGUGag-3') and a non-targeting siRNA (siNEG) as negative control from AMBION (Life Technologies LTD, Waltham, MA). The Lipofectamine RNAiMAX reagent (Thermo Fisher Scientific, Waltham, MA) was used according to manufacturer's instructions. Transfected cells were then cultured for 24-48 hours depending on the experimental setup and analysis of silencing specificity verified by Western Blot. For transfection of plasmid constructs TransIT®-LT1 Reagent (Mirus Bio LLC, Madison, WI) has been used according to manufacturer's instructions.

### Luciferase reporter assay

GBM cells were transfected using a protocol for transient transfection of adherent cells using TransIT®-LT1 Transfection Reagent (Mirus Bio LLC, Madison, WI) with BAT-luciferase reporter construct (BAT-lux) (Addgene plasmid # 20890). This consists of seven TCF/LEF-binding sites upstream of a 0.13-kb fragment containing the minimal promoter-TATA box of the gene siamois driving the expression of Firefly luciferase reporter gene 29. To over-express TCF4 inhibitory isoforms (TCF4E) we used the plasmid pcDNA3.1-TCF4E (Addgene plasmid # 32738). Luciferase experiments were set as follows: at day 1 cells were plated at 2x10^5^ per well and at day 2 transfected with pcDNA3.1-TCF4E or pcDNA3.1. At day 3, cells were transfected with BAT-lux and a pMAX-GFP plasmid as a proper control of transfection efficacy and for normalization purposes. At day 4 cells were treated or not with Wnt3a (30ng/ml) and then at day 5 solubilized in passive lysis buffer (PLB, Promega, Madison, WI) and luciferase activity measured by a Victor 3 multi-well plate reader (Perkin Elmer, Waltham, MA). The same experimental setup was used for luciferase reporter assays after *TCF7* and *TCF7L2* gene silencing (day2). Reporter activation values are expressed as relative light units (RLUs).

### ChIP-droplet digital PCR (ddPCR)

To investigate the molecular binding of transcription factors to specific DNA sequences, we set up ChIP experiments as follows: first, we transfected GBM cells cultured under hypoxic or normoxic conditions with the BAT-lux reporter. The day after, cells were treated or not with Wnt3a and/or silenced for TCF1 (siNEG as negative control). The next day, cells were fixed with formaldehyde, lysated and sonicated as described above. Then, immunoprecipitation was performed using HIF-1α antibody (irrelevant IgG antibodies have been used as isotype control). Purification of plasmid-DNA was performed by phenol/chloroform extraction. Input samples have been also retrieved for subsequent data normalization. To analyze DNA sequences bound to HIF-1α we set up ddPCR experiments using EvaGreen Digital PCR Supermix (Bio-Rad, Hercules, CA) and primers able to amplify the 7xTCF binding sites DNA sequence (Table S5). Then, each sample was emulsionated and the obtained droplets were processed in a standard thermal cycler according to manufacturer's instructions. After the amplification, droplets generated for each sample (Input, IgGs, ChIP samples) were processed in the QX200 Droplet Digital PCR (ddPCR™) System (Bio-Rad, Hercules, CA). DNA quantity of each ChIP sample was measured as DNA copies/µl and normalized for the appropriate Input sample, obtaining the enrichment over input ratio plotted on bar graphs. ddPCR experiments using EvaGreen Digital PCR Supermix (Bio-Rad, Hercules, CA) were set up also for the amplification “neuronal genes” in order to compare their expression levels in H/N GBM cells ±Wnt3a. Primers used are listed in Supplementary Table S5 and specificity confirmed by Human BLAT Search (http://genome.ucsc.edu). Relative mRNA quantities for each gene was measured as RNA copies/µl, normalized to GUSB mRNA and plotted on bar graph or level plot.

### Immunohistochemistry

TCF1, HIF-1α and β-III-tubulin staining was performed on 5μm sections of paraffin embedded GBM specimens with standard procedures. Briefly, sections were re-hydrated and then antigen retrieval was performed by incubation with citrate buffer 0.01M pH6 at 95°C. After saturation with normal serum, slides were incubated with primary antibodies: anti-TCF7 (TCF1; 1:50, Sigma-Aldrich, St. Louis, MO, #WH0006932M1), anti-TCF7L2 (TCF4; 1:100, Sigma-Aldrich #SAB1404454), anti-HIF-1α (1:100, Sigma-Aldrich, #HPA001275) and anti-β-III tubulin (TUJ1; 1: 500, Covance, Princeton, NJ, #MMS-435P). After incubation, sections were washed and incubated with species-specific biotin-conjugated secondary antibodies (Vector Laboratories Inc., Burlingame, CA). TCF1, HIF-1α and β-III-tubulin expression was revealed by using the Dako Liquid DAB+ Substrate Chromogen System (Dako, Glostrup, Denmark) according to manufacturer's guidelines. Tissues were counterstained with Meyer's Hematoxylin and images acquired with a Zeiss Imager M1 microscope (Carl Zeiss, Oberkochen, Germany). The specificity of each staining procedure was confirmed by replacing the primary antibodies with an Isotype control. The expression levels of TCF1, TCF4 and HIF-1α were scored using a combined method accounting for both the staining intensity and the percentage of positive stained cells. The resulting combined score was calculated as the multiplication of both the percentage of positive cells (0-6) and the staining intensity (0-3).

### Statistical analyses

Graphs and associated statistical analyses were generated using Graph Pad Prism 7.03 (GraphPad, La Jolla, CA). All data in bar graphs are presented as mean ± standard error of the mean (S.E.M.). Statistical significance was measured by one-way ANOVA with Newman-Keuls multiple comparison post-test (for more than two comparisons) and paired *t*-test (comparison of two groups): *p<0.05, **p<0.01, ***p<0.001, ****p<0.0001. For all graphs, asterisks over brackets indicate a significant difference with another variable as indicated and asterisks over bars indicate a significant difference with the control group.

For neurosphere limiting dilution assay Analysis of Covariance (ANCOVA) has been performed in order to compare slopes of linear regressions.

Integration of IHC data has been obtained by applying Principal Component Analysis to IHC scores (Partek Genomic Suite Software v.7.0, Partek Inc., St.Louis, MO) and contingency tables analyzed by chi-square test. Survival analyses were performed by generating Kaplan Meier survival curves and significance calculated by log-rank (Mantel-Cox) test. In particular, in order to identify the proper cutoff values for selecting comparison groups for survival, multivariate Cox analysis (Wald test) was applied. The comparison between the “0-6 genes” and “>7 genes” groups resulted as the unique cutoff demonstrating statistical significance.

## Results

### Wnt signaling activation distinctively affects GBM stem cell phenotype, depending on microenvironmental oxygen

**Figure 1:**
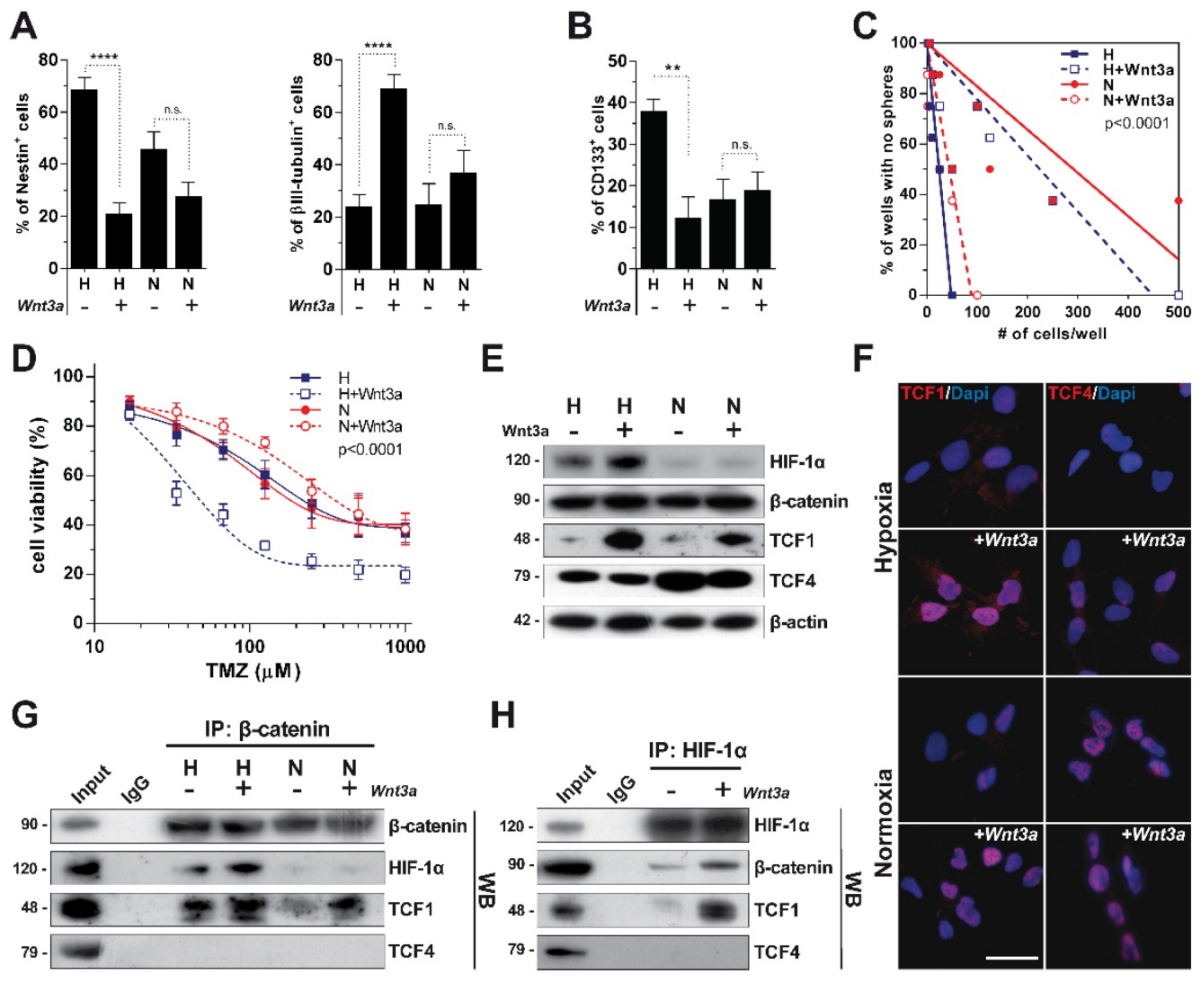
** Microenvironmental oxygen tension modulates a Wnt-dependent differentiation of GBM cells and the expression of Wnt-signaling co-factors.** (**A**) Percentage of Nestin^+^ (left panel) and βIII-tubulin^+^ (right panel) GBM cells (n=12) cultured in hypoxia (H) or normoxia (N) and acutely exposed to Wnt3a (30ng/ml) for 96 hours. (**B**) Percentage of CD133^+^ GBM cells (n=5) under the same conditions as in (**A**). (**C**) Limiting dilution analysis of the frequency of GBM cells able to generate neurospheres after exposure to H (blue lines), N (red lines) and Wnt3a stimulation in both conditions (dotted lines). (**D**) Dose-response curves of TMZ (48h) in Wnt3a pre-treated (96h) or control (dotted and solid curves, respectively) GBM cells (n=3) in H (blue) or N (red) conditions. (**E**) Representative (HuTuP53) WB analysis of HIF-1α and the Wnt pathway effectors β-catenin, TCF1 and TCF4 in cells under H/N±Wnt3a (30ng/ml; 24h). β-actin was used as a loading control. (**F**) Representative immunofluorescence analysis of TCF1 and TCF4 expression (red) in GBM cells (HuTuP13) cultured and treated as in (**A**). Nuclei were counterstained with DAPI (blue). Original magnification 40x; bar=50µm. (**G-H**) Representative WB analysis of GBM cell lysates (HuTuP53) treated as in (**E**), immunoprecipitated by β-catenin (**G**) or HIF-1α (**H**) antibodies and then stained with β-catenin, HIF-1α, TCF1 and TCF4 antibodies. In (**H**) only GBM cells exposed to H are shown. Molecular weights in kDa are reported near WB panels. In all graphs, mean ± S.E.M. of at least 3 independent experiments is shown. ****p<0.0001; *p<0.05; H: hypoxia; IP: immunoprecipitation; N: normoxia; n.s.: not significant; TMZ: temozolomide; WB: western blot.

Starting from our previous observation that Wnt activation promotes a dramatic neuronal differentiation of GBM cells under hypoxia 19, we sought to investigate if these Wnt-mediated effects could be modulated by the oxygen tensions to whom cells are exposed. To this end, we treated patient-derived primary GBM cells with recombinant Wnt3a either in hypoxia or in normoxic conditions and assessed their response in terms of stemness/differentiation status. As expected, Wnt signaling activation under hypoxia promoted a significant reduction of the NSC markers Nestin and CD133, together with a strong acquisition of neuronal traits (Figure 1A-B and Figure S1A). Conversely, normoxia, besides decreasing the expression of NSC markers by itself as previously reported 30, de-sensitized GBM cells to the Wnt-induced phenotypic shift (Figure 1A-B and Figure S1A). As a functional validation of these effects, hypoxic Wnt signalling activation significantly reduced both the GBM stem cell frequency and their ability to form neurospheres with normoxic conditions even enhancing their self-renewing properties (Figure 1C and Figure S1B-C). Since more differentiated GBM cells display higher sensitivity to alkylating agents-based chemotherapy 11, 31, 32, we treated control and Wnt3a pre-treated cells with TMZ and showed that hypoxic Wnt-stimulated GBM cells were significantly sensitized to TMZ treatment (Figure 1D).

### Oxygen availability differentially modulates TCF1 and TCF4 levels

In order to identify a molecular rationale underlying these effects, we evaluated the protein levels of the β-catenin transcriptional co-factors TCF1 and TCF4 upon Wnt stimulation and their potential correlation with HIF-1α stabilization. Wnt administration strongly induced TCF1 expression. In contrast, TCF4 showed increased expression only in normoxia (Figure 1E-F). Of note, multiple alternative splicing products have been described for TCF4 33, but only hMW TCF4 isoforms were abundantly expressed in GBM cells (Figure 1E), with smaller forms being barely detectable in our setting (Figure S1D, left).

To evaluate how these transcriptional co-factors interact in different microenvironmental conditions, we immunoprecipitated β-catenin and HIF-1α in GBM cells treated with Wnt3a at different oxygen tensions. TCF1 participated in the formation of a HIF-1α/β-catenin transcriptional complex (Figure 1G-H). On the other hand, hMW TCF4 isoforms were not able to bind neither HIF-1α nor β-catenin (Figure 1G-H), which rather interacted with smaller TCF4 splicing products (Figure S1D, right). These data suggest that a transcriptional complex containing TCF1, β-catenin and HIF-1α can assemble upon hypoxic Wnt signaling stimulation and might be involved in sustaining the reported neuronal differentiation of GBM stem cells. Moreover, since hMW TCF4 isoforms do not interact with β-catenin (Figure 1G) and have been described to possess inhibitory effects on gene transcription 34, we hypothesized their higher expression in normoxia could hamper Wnt activation and mild neuronal differentiation of cells.

### Hypoxia cooperates with Wnt pathway to regulate the transcriptional milieu of GBM cells

**Figure 2:**
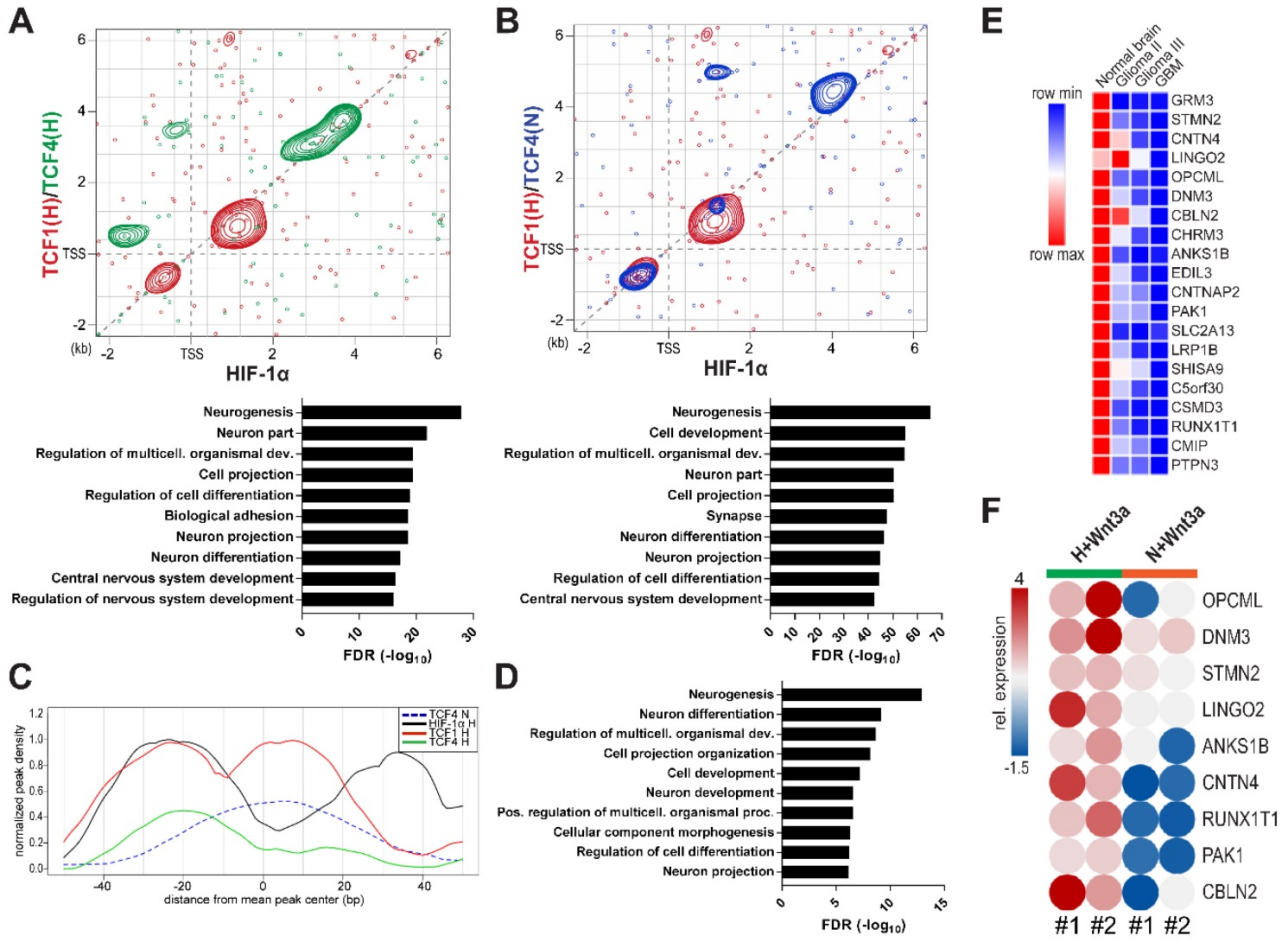
** A differential co-operation between HIF-1α, TCF1 and TCF4 controls peculiar GBM transcriptional features.** (**A**-**B**) Scatter plots representing the peak localization around gene transcriptional starting sites (TSS) of HIF-1α and TCF1 (red) in H or TCF4 under H (**A**, green) or N (**B**, blue) after Wnt3a exposure; areas with higher concentration of peaks are evidenced by contour lines. In bottom panels, GO analysis of genes bound by HIF-1α/TCF1 and TCF4 in both H/N is reported. (**C**) Cumulative ChIP-seq profiles of analyzed transcription factors around peaks endowed with a HIF-1α (black line)/TCF1 (red line) co-localization signal under H. TCF4 binding in H and N microenvironments is shown by green and blue lines, respectively. (**D**) GO analysis obtained from genes selected to contain a HIF-1α/TCF1 co-localization binding pattern as described in (**C**). (**E**) Level plot representing the median fold change of the top 20 genes down-regulated in GBM relative to normal brain samples from the GSE4290 dataset 27 and showing a co-localization of HIF-1α, TCF1 (in H) and TCF4 (in N). (**F**) Levelplot showing the relative mRNA expression of selected genes from (**E**) in Wnt3a-treated (n=2; 30ng/ml) cells under H/N (p<0.0001 by global two-way Anova test). FDR: false discovery rate; GO: gene ontology; H: hypoxia; N: normoxia; TSS: transcriptional starting site.

With the aim of assessing the impact of HIF-1α, TCF1 and TCF4 in controlling the transcriptional landscape of GBM cells, we immunoprecipitated all these factors in different microenvironmental conditions and sequenced their cross-linked genomic regions by a ChIP-seq approach. Examination of their binding patterns in hypoxic samples revealed areas of co-binding between HIF-1α and TCFs. However, we disclosed a differential genomic distribution of TCF4, depending on microenvironmental oxygen (Figure S2A-B). HIF-1α/TCF1 co-immunoprecipitated sequences in normoxia did not produced interpretable high-quality peak calls, thus excluding them from further analysis (data not shown). Importantly, TCFs and HIF-1α DNA binding motifs were enriched among the immunoprecipitation-derived fragments, thus confirming the specificity of antibodies used (Figure S2C). When we focused on genomic regions around the Transcriptional Starting Site (TSS) of genes (-2kb - +6kb), we confirmed TCF1 and TCF4 intrinsic transcriptional function in regulating genes that control metabolic, trafficking and transcriptional processes under hypoxic conditions (Figure S2D-E). A deeper characterization of the transcriptional cooperation between TCF1 and HIF-1α disclosed their tendency to co-localize within ±2kb near gene TSSs (Figure 2A, red contours) instead of TCF4, which rather co-localizes with HIF-1α on more distal sequences (Figure 2A, green contours). Gene Ontology (GO) analysis of the gene lists retrieved from the sum of these identified regions revealed that these factors potentially act together in controlling the expression of genes involved in neurogenesis and neuron differentiation (Figure 2A, bottom graph). Furthermore, at higher oxygen levels, TCF4 was located onto the same genomic sequences shown to be controlled by HIF-1α and TCF1 under hypoxia, (Figure 2B, blue contours), thus sustaining its putative role as regulator of neuronal differentiation genes also in these conditions (Figure 2B, bottom graph). Since in normoxia we never observed a Wnt-induced neuronal differentiation of GBM cells (Figure 1A-C and Figure S1A-C)), these results further point to TCF4 as a transcriptional inhibitor of this process.

### A HIF-1α/Wnt signaling-controlled gene signature involved in neuronal differentiation is progressively switched off in high-grade gliomas

By increasing analysis resolution, we retrieved a more detailed localization of HIF-1α and TCF1 on the genome of GBM cells. In particular, we observed that a subset of genes (n=105), showing the concurrent binding of both HIF-1α and TCF1, were characterized by a 40bp region to the left of the mean peak center containing HIF-1α and TCF1 overlapping binding sites. Moreover, these were flanked by two consecutive downstream regions (~20bp/each) displaying TCF1 and HIF-1α specific bindings, respectively (Figure 2C). Interestingly, GO analysis of the gene list (n=99) retrieved from the intersection of genes containing this 80bp regulatory window with genes potentially controlled by TCF4 in normoxia (Figure S3A) showed their significant involvement in neurogenesis and neural cell differentiation processes (Figure 2D). This gene list was further used to generate a transcriptional signature (91 genes; Table S3) able to partially discriminate normal brain samples from gliomas of different grades in the GSE4290 dataset 27 using an unsupervised analytical approach (Figure S3B). In addition, a deepened expression analysis of genes belonging to this signature evidenced that genes down-regulated in GBM samples relative to normal brain (n=54) are implicated in neurogenesis and structural and phenotypic maturation of neurons. On the contrary, up-regulated genes (n=37) are more correlated to basic metabolic and survival processes (Figure S3C-D). Of note, top-ranked down-regulated genes (n=20 on the basis of their fold change) were characterized by their progressive shutdown with increasing glioma grade, thus suggesting their potential role in weakening tumor aggressiveness (Figure 2E). Confirming their critical involvement in the process of neuronal differentiation, we found the expression of 13/20 of these genes as highly over-expressed in fetal neuron LUHMES cells that have been differentiated into mature dopamine-like neurons 35 relative to their undifferentiated counterpart (Figure S3E-F).

In order to transpose this information to our experimental setting, we evaluated the transcriptional levels of these genes upon hypoxic/normoxic Wnt signaling stimulation and showed their striking Wnt3a-dependent increase only in hypoxia (Figure 2F). These data confirm the functional existence of a transcriptional juxtaposition between the HIF-1α/TCF1 complex and TCF4, depending on microenvironmental oxygen tension.

### TCF4 exerts a transcriptional inhibitory function on neuronal differentiation, which is hampered by HIF-1α stabilization

**Figure 3:**
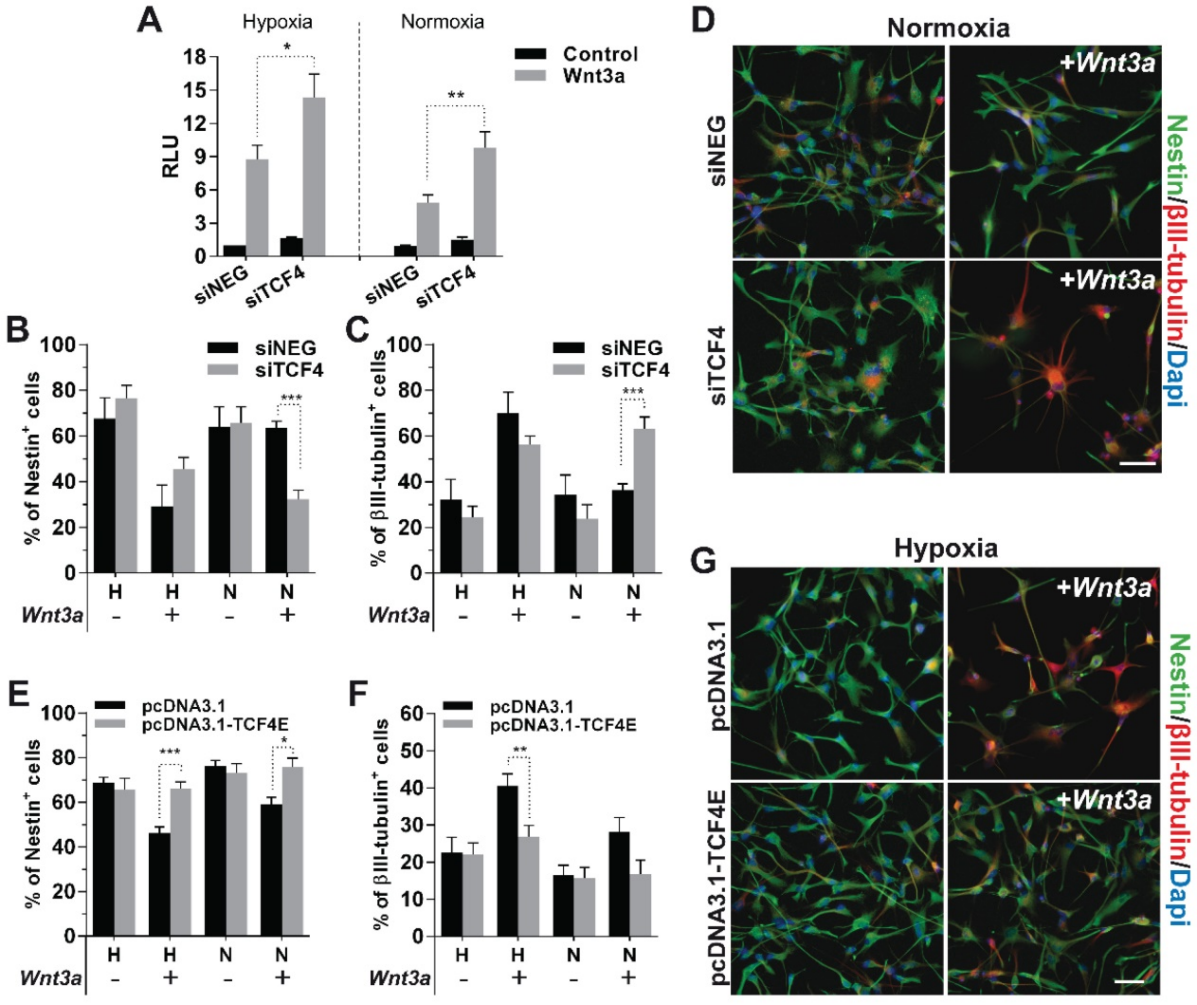
** TCF4 exerts a transcriptional inhibitory function on neuronal differentiation.** (**A**) Bar graph showing the relative Wnt signaling activation in H/N TCF4-silenced GBM cells (n=3) ±Wnt3a (30ng/ml for 24h) by using the luciferase reporter construct BAT-lux. (**B**-**C**) Percentage of Nestin^+^ (**B**) and βIII-tubulin^+^ (**C**) TCF4-silenced cells (n=5) treated (+) or not (-) with Wnt3a (30ng/ml for 4d) under H/N conditions by immunofluorescence. (**D**) Representative immunofluorescence images of cells (HuTuP13 under N) summarized in (**B**, **C**) which have stained with Nestin (green) and βIII-tubulin (red) antibodies. (**E**-**F**) Percentage of Nestin^+^ (**E**) and βIII-tubulin^+^ (**F**) TCF4E-over-expressing GBM cells (n=12) treated (+) or not (-) with Wnt3a (30ng/ml for 4d) under H/N conditions by immunofluorescence. (**G**) Representative immunofluorescence images of cells (HuTuP53 under H) summarized in (**E**-**F**). In (**D**, **G**) original magnification 20x; bar=50µm. In all images cell nuclei have been counterstained with Dapi (blue). ***p<0.001; **p<0.01; *p<0.05; d: days; H: hypoxia; N: normoxia; RLU: relative light units.

Analysis of ChIP-seq data strongly supports the idea that, in GBM cells, TCF4 mainly acts as a transcriptional inhibitor of the Wnt-driven differentiation capability. In order to verify this hypothesis, we silenced TCF4 and exposed GBM cells to Wnt3a stimulation in either hypoxic or normoxic conditions. TCF4 knockdown (Figure S4A-B) significantly increased the activation of the luciferase-based Wnt pathway reporter construct BAT-lux (Figure 3A). Moreover, TCF4 suppression was sufficient to functionally release a Wnt-induced neuronal differentiation also in normoxic conditions (Figure 3B-D and Figure S4C). To finally highlight the inhibitory role of TCF4 on differentiation, we over-expressed a hMW TCF4 isoform (75kDa; TCF4E; Figure S4D-E) in hypoxia which fully prevented the Wnt3a-mediated neuronal differentiation (Figure 3E-G), nevertheless only partially affecting the phenotype of normoxic cells (Figure S4F).

**Figure 4:**
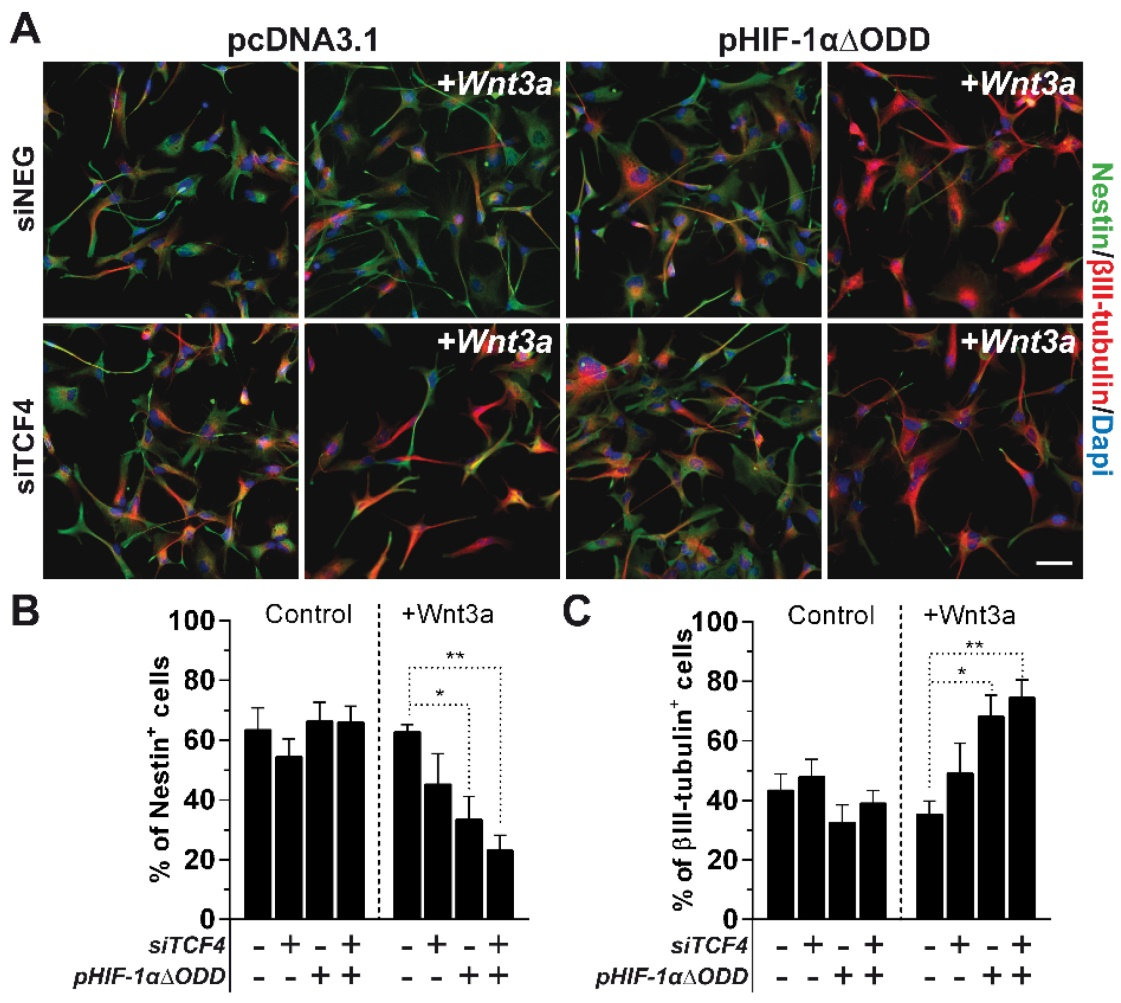
** Microenvironmental regulation of TCF4 expression is a non-redundant mechanism to control GBM cell differentiation.** (**A**) Representative immunofluorescence images of normoxic GBM cells (HuTuP53) in which TCF4 have been silenced, HIF-1αΔODD over-expressed or both and then treated with Wnt3a (30ng/ml for 4d). GBM cells transfected with siNEG or pcDNA3.1 or both have been used as proper controls. Cells have been stained with Nestin (green) and βIII-tubulin (red) antibodies and counterstained with Dapi (blue). Original magnification 20x; bar=50µm. (**B**-**C**) Bar graphs reporting the quantification of Nestin^+^ (**B**) and βIII-tubulin^+^ (**C**) GBM cells (n=8) as in (**A**). **p<0.01; *p<0.05; d: days.

In the end, we tested whether the sole stabilization of HIF-1α, rather than a weakened TCF4 expression observed under hypoxia (Figure 1E-F), could be sufficient in counteracting the inhibitory role of TCF4 against GBM cell differentiation. In this context, the exogenous stabilization of a truncated form of HIF-1α, deleted for its Oxygen Dependent Domain (ODD) 31, 36, namely HIF-1αΔODD (Figure S5), significantly impaired the described TCF4-dependent block of differentiation in normoxic GBM cells, thus allowing their partial Wnt-dependent acquisition of neuronal traits (Figure 4A-C). However, the concomitant silencing of TCF4 in a HIF-1α over-activated environment further enhanced this pro-neuronal differentiating effect (Figure 4A-C). These latter data demonstrate that a lowered TCF4 expression under hypoxic condition is a crucial non-redundant mechanism ensuring the proper translation of the Wnt stimulus into an efficient neuronal commitment of GBM cells.

These results clearly show that a multi-faceted intervention of hypoxia in both the control of TCF4 expression levels and the recruitment of HIF-1α into the β-catenin/TCF1 transcriptional complex is a desired requirement for GBM cells in order to engage a reminiscent pro-neuronal transcriptional program.

### TCF1 is the master regulator of Wnt-induced neuronal differentiation

**Figure 5:**
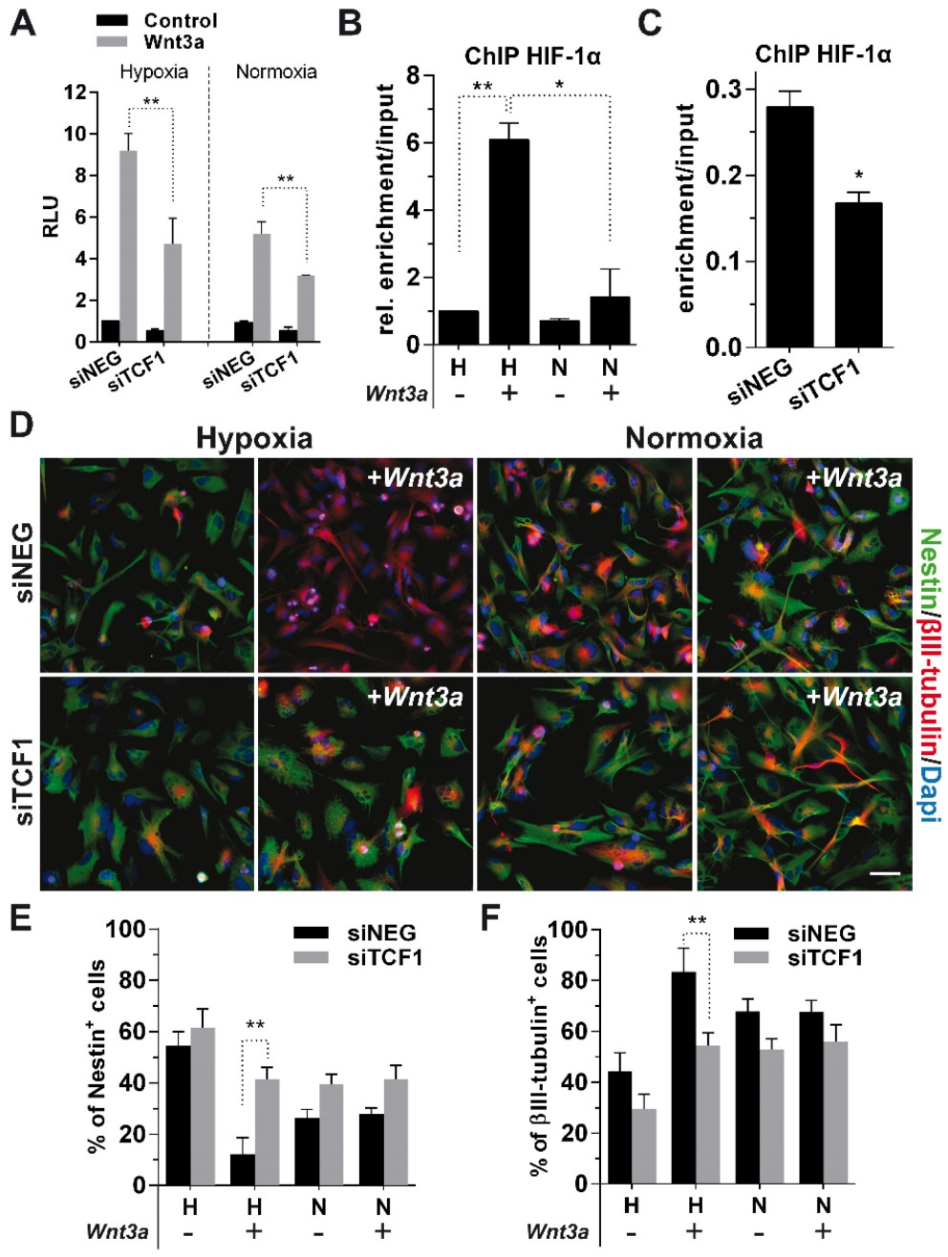
** TCF1 is the major driver of neuronal differentiation in GBM cells.** (**A**) Bar graph showing relative Wnt signaling activation in H/N TCF1-silenced GBM cells (n=3) ±Wnt3a (30ng/ml for 24h) by using the luciferase reporter construct BAT-lux. (**B**) Graph representing the relative enrichment by ddPCR of TCF/LEF binding site sequences in H/N GBM cells (n=3) transfected with BAT-lux ±Wnt3a (30ng/ml; 24h) and immunoprecipitated for HIF-1α. (**C**) Graph representing relative ddPCR amplification of TCF/LEF binding site sequences in hypoxic TCF1-silenced or control cells (n=3) transfected with BAT-lux ±Wnt3a (30ng/ml; 24h) and immunoprecipitated for HIF-1α. In (**B-C**) the relative enrichment normalized to Input samples is reported. (**D**) Representative immunofluorescence images of H/N TCF1-silenced GBM cells (HuTuP13) ±Wnt3a (30ng/ml for 4d) stained with Nestin (green) and βIII-tubulin (red) antibodies and counterstained with Dapi (blue). Original magnification 20x; bar=50µm. (**E-F**) Bar graphs reporting the quantification of Nestin^+^ (**E**) and βIII-tubulin^+^ (**F**) GBM cells (n=6) as in (**D**). **p<0.01; *p<0.05; ChIP: chromatin immunoprecipitation; d: days; ddPCR: droplet digital PCR; h: hour; H: hypoxia; N: normoxia; RLU: relative light units.

To confirm the suggested role of TCF1 in sustaining GBM cell differentiation, we silenced its expression in Wnt3a-stimulated cells (Figure S4A-B), which resulted in a significant weakening of the activation of BAT-lux reporter upon Wnt stimulation (Figure 5A). Since we previously showed that TCF1 and HIF-1α both participate in the formation of a Wnt-dependent transcriptional complex seating on peculiar DNA sequences (Figure 2A-C), we characterized the mechanism by which HIF-1α is recruited onto the TCF consensus. To this aim, we transfected GBM cells with the BAT-lux reporter construct, which contains 7xTCF/LEF consensus sequences 29, immunoprecipitated HIF-1α and then performed a ChIP-droplet digital PCR (Chip-ddPCR) with specific primers flanking the Wnt-responsive sequences in the plasmid (Figure S6A). HIF-1α, besides placing in the promotorial region of its target gene *CAIX* (Figure S6B), was clearly located onto TCF/LEF consensus sequences upon Wnt3a stimulation (Figure 5B). Importantly, TCF1 silencing partially abrogated this binding (Figure 5C), suggesting that HIF-1α is recruited on Wnt consensus sequences by a transcriptional complex containing TCFs co-factors.

Data obtained so far, clearly point at the interaction between HIF-1α and TCF1 as the fundamental mechanism sustaining the Wnt-mediated neuronal differentiation of GBM cells. Indeed, phenotypic analyses unambiguously showed that TCF1-silenced cells underwent a complete block of their neuronal differentiation potential (Figure 5D-F), thus demonstrating the pivotal function of TCF1 as an essential mediator of the neuronal differentiation process in hypoxic GBM cells.

### TCF1 and HIF-1α levels positively correlate with neuronal differentiation in gliomas

**Figure 6:**
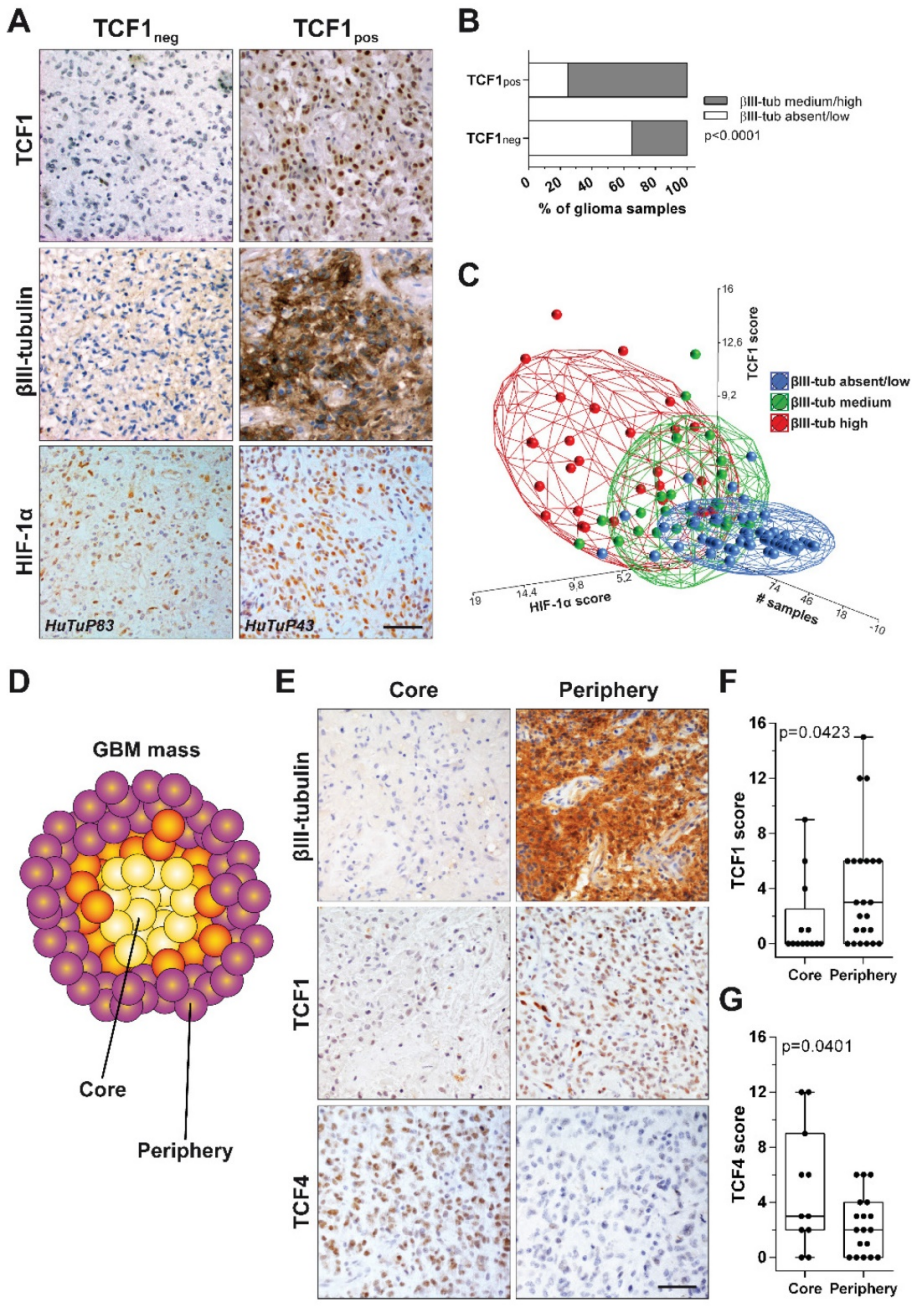
** TCF1 and HIF-1α levels positively correlate with neuronal differentiation in GBM tissues.** (**A**) Representative IHC staining of TCF1 (up), β-III tubulin (middle) and HIF-1α (bottom) protein levels in a cohort of 128 glioma specimens. Original magnification 40x; bar=50µm. (**B**) Bar graph showing a significant enrichment (by chi-square analysis) of βIII-tubulin expressing (medium/high intensity) biopsies in TCF1_neg_ and TCF1_pos_ glioma samples. (**C**) PCA showing an existing positive correlation between TCF1 (score), HIF-1α (score) and βIII-tubulin (blue: absent/low; green: medium; red: high-intensity) in our cohort of glioma samples. (**D**) Cartoon representing the spatial distribution of Core and Periphery derived biopsies throughout the GBM mass. (**E**) Representative IHC staining of β-III tubulin (up), TCF1 (middle) and TCF4 (bottom) in a cohort of GBM patients sampled for tumor Core and Periphery. Original magnification 40x; bar=50µm. (**F-G**) Box plots reporting the quantification of TCF1 (**F**) and TCF4 (**G**) scores in GBM Core and Periphery (p value calculated by Mann-Whitney). IHC: immunohistochemistry; neg: negative; PCA: principal components analysis; pos: positive.

In order to test the clinical relevance of our findings, we stained by immunohistochemistry (IHC) 128 samples retrieved from 87 glioma patients for the expression of HIF-1α, TCF1, TCF4 and the neuronal marker βIII-tubulin. A preliminary comparison between TCF1^-^ (score 0) and TCF1^+^ (score 1-15) samples disclosed this latter subgroup as characterized by a significant enriched expression of βIII-tubulin (Figure 6A-B). Moreover, by performing a multi-correlation analysis, we intriguingly found that: i) samples harboring a relatively low expression of HIF-1α and a low/absent staining for TCF1 showed a restricted βIII-tubulin expression; ii) low HIF-1α, but high positivity for TCF1 were found in samples characterized by an intermediate expression of βIII-tubulin; iii) glioma samples displaying high expression levels of both HIF-1α and TCF1 were also endowed with the most intense βIII-tubulin staining (Figure 6A-C). Supporting our previous *in vitro* findings, the distribution of TCF4 scores among GBM samples suggested their negative correlation with both HIF-1α (Figure S7A) and βIII-tubulin (Figure S7B-C). These results further correlate the co-expression of TCF1 and HIF-1α with a neuronal-differentiated phenotype in human gliomas.

**Figure 7:**
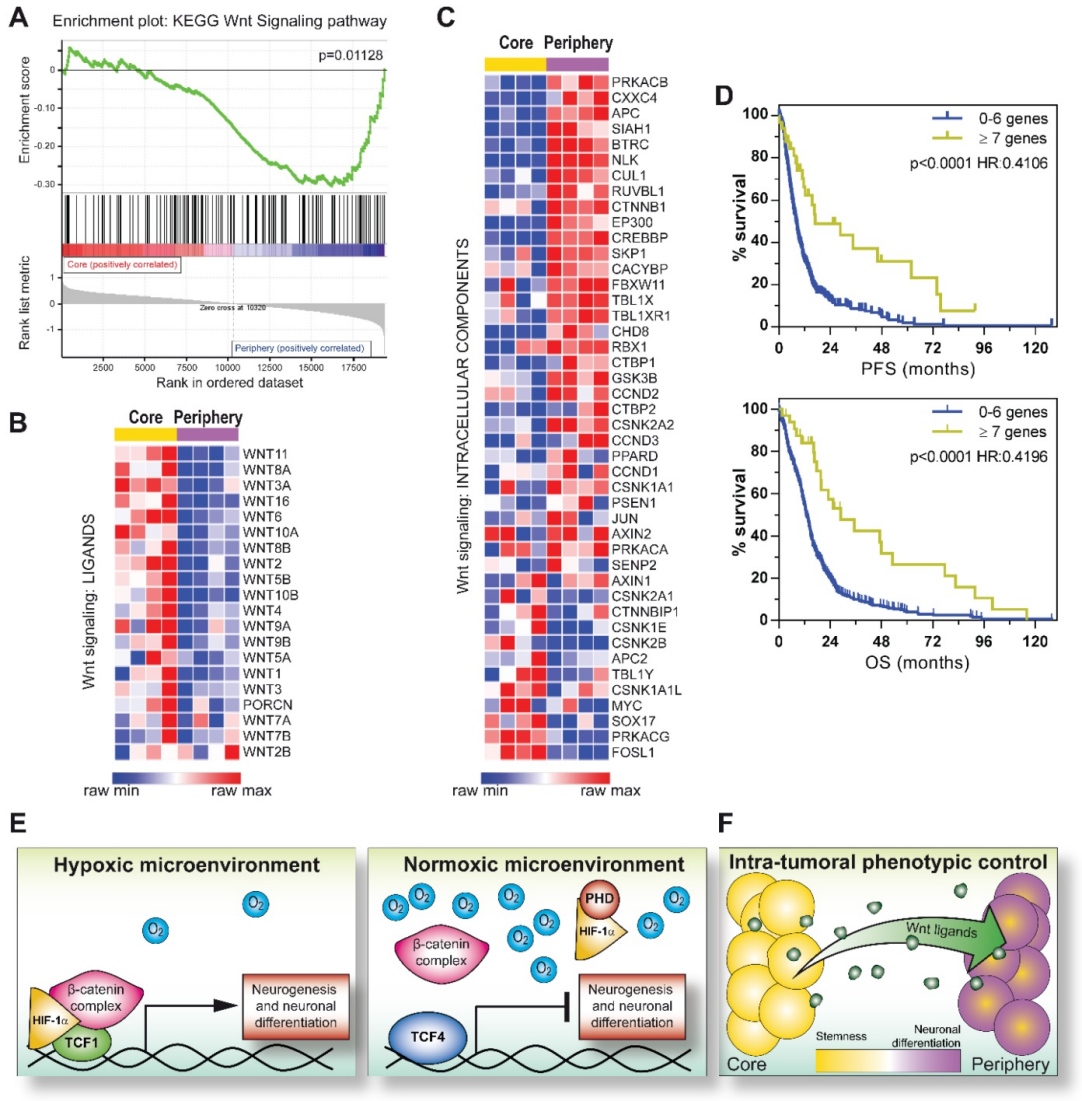
** Intra-tumoral heterogeneity is sustained by a different topographical activation of Wnt signaling in the GBM mass.** (**A**) GSEA of differentially expressed genes between Core and Periphery showing significant enrichment for KEGG Wnt signaling pathway gene set. (**B-C**) Level plots reporting the differential expression of Wnt signaling ligands (**B**) or Wnt signaling intracellular components (**C**) between GBM Core and Periphery. (**D**) Kaplan Meier curves showing the impact of the number of over-expressed Wnt genes (with z-score≥1.5) on GBM patient outcome (TCGA dataset 28) in terms of PFS (top) and OS (bottom). p calculated by log-rank (Mantel-Cox) test. HR calculated as [risk of patients with ≥ 7 Wnt genes (n=33; z-score≥1.5)]/[risk of patients with 0-6 Wnt genes (n=486; z-score≥1.5)]. (**E**) Models summarizing the proposed *in vitro* mechanisms that controls GBM cell phenotype. Hypoxia contributes to a Wnt dependent pro-differentiative transcriptional program through the formation of a HIF-1α/TCF1 complex (left). hMW TCF4 isoforms exert a transcriptional inhibitory function on HIF-1α/TCF1-dependent neuronal differentiation (right). (**F**) Intra-tumoral phenotypic heterogeneity reflects a differential topographical expression of Wnt signaling components. GSEA: gene set enrichment analysis; hMW: high molecular weight; HR: hazard ratio; OS: overall survival; PFS: progression free survival.

In this context, since we previously demonstrated that GBM tumors are spatially organized in multiple concentric layers characterized by peculiar phenotypic and functional cellular identities 11, 37, 38, we evaluated if a Wnt-dependent control of cell differentiation could sustain GBM intra-tumoral heterogeneity. In particular, IHC staining of spatially-distributed biopsies resected from 24 GBM tumors according to this concentric model (Figure 6D), showed that peripheral tumor tissues were characterized by high βIII-tubulin expression and a significantly higher positivity for TCF1. Core-derived tumor tissues rather displayed high levels of TCF4 (Figure 6E-G). Moreover, by comparing their transcriptional profiles, we identified 1161 differentially expressed genes discriminating the core from the tumor periphery by an unsupervised analytical approach (Figure S8A). Interestingly, although both series of samples (from tumor core and periphery) displayed a similar protein stabilization of HIF-1α (Figure S8B), the activation of transcriptional signatures related to hypoxia or HIF-1α was only observed in core samples. More peripheral regions of the tumor were instead negatively enriched for hypoxic signatures and even characterized by oxidative phosphorylation dependent transcripts (Figure S8C-D). These data prompted us to hypothesize that, in the peripheral/neuronal differentiated GBM samples, HIF-1α may primarily act as a co-factor of the Wnt pathway, rather than activating its recognized target genes, thus actively participating in the enhancement of the Wnt signaling-dependent neuronal differentiation process. Indeed, GSEA additionally highlighted a clear-cut transcriptional enrichment of Wnt signaling pathway in GBM cells isolated from the tumor periphery (Figure 7A), thus corroborating the involvement of Wnt signaling in the selective acquisition of neuronal traits in specific cell subpopulations (Figure 6E-F). Surprisingly, we identified core residing GBM cells as the major source of Wnt-ligands expression (Figure 7B) and thus potentially able to stimulate Wnt pathway activation in peripheral cells. Indeed, these seem to be more prone to engage an effective Wnt signaling activation due to an intrinsic over-expression of several intracellular pathway components (Figure 7C and Figure S9A-B). Conversely, Wnt receptors and members of the cell polarity or calcium (Wnt-dependent) pathways were not differentially modulated (Figure S9C-E). Further confirming these results, we show that genes belonging to a peculiar transcriptional signature (genes co-regulated by HIF-1α/TCF1 in hypoxia and by TCF4 in normoxia; Figure 2C-E and Figure S3A) that we demonstrated to be: i) progressively switched off in GBM (Figure 2E and Figure S3B); ii) significantly upregulated in GBM cells exposed to hypoxic Wnt3a stimulation (Figure 2F); iii) highly expressed in finally differentiated dopaminergic normal neurons (Figure S3E-F), are significantly over-expressed (60/84) also in these neuronal-differentiated peripheral regions of GBM tumors (Figure S9F).

With the aim of assessing whether the observed imbalance of Wnt ligands/intracellular effectors across different regions of the same mass could affect GBM patient prognosis, we looked for tumors with any of the Wnt intracellular pathway components (Figure 7C) over-representation in the TCGA GBM tumor dataset 28. GBM patients displaying up-regulation of 7 or more “Wnt genes” (the selected 44 genes listed in the levelplot of Figure 7C; z score≥1.5), showed a significant prolonged progression free and overall survival compared to GBM tumors over-expressing a limited number (0-6) of these genes (Figure 7D), indicating once again that the activation of Wnt signaling in these tumors, even if subordinated by peculiar microenvironmental conditions, may sustain their differentiated and less aggressive phenotype.

## Discussion

GBM represents a dramatic challenge for clinicians and researchers, with radio- and chemotherapy being often administered to GBM patients only as palliative treatments 39. In this context, a subpopulation of CSCs has been demonstrated to sustain peculiar hallmarks of cancer in GBM tumors 40-42. For this reason, the further application of the principles of stem-cell biology to the study of GBM promises to unveil potential major mechanisms supporting its severity 43. Based on this concept, the study of the microenvironmental stimuli able to regulate CSC survival and the (dis)organized growth of GBM tumors is particularly relevant 2, 30, 44. Recent literature points out the importance of transcriptional machineries in deciding cell fate through a microenvironment-driven tight modulation of their gene expression profiles 45-48. That is why we focused our strengths in dissecting the role of β-catenin/TCFs transcriptional complex in inducing a reminiscent neuronal differentiation of GBM cells and its molecular relationship with the major sensor of the hypoxic stimulus, HIF-1α. In GBM, the role of the canonical Wnt signaling-dependent transcriptional functions, through the β-catenin/TCFs complex, has been extensively debated 19, 49-52. However, the specific interactions of different co-factors and how they respond to external microenvironmental stimuli has never been fully elucidated.

Here, we interestingly describe a transcriptional inhibitory function of TCF4, which seems to be the major antagonist of the HIF-1α/TCF1-mediated neuronal differentiation in GBM cells (Figure 7E). In this context, it has been previously reported that hMW TCF4 isoforms (i.e. TCF4E), besides containing two consecutive HMG-box DNA binding domains, a Nuclear Localization Signal (NLS) and an N-terminal β-catenin binding domain (BCBD), are characterized by the inclusion of a longer C-terminal exon 17 harboring two binding sites for CtBP co-repressors. For these characteristics, they have been suggested to exert inhibitory functions on gene transcription 33, 34, 53. Of note, GBM cells show a very high expression of hMW TCF4 isoforms, although a fine identification of the specific alternative splicing-dependent TCF4 products has not been investigated in our study. Moreover, the known Glycogen synthase kinase 3 (GSK3) inhibitor LiCl has previously demonstrated to induce a GSK3-independent transcriptional shift from hMW to shorter TCF4 isoforms in endothelial cells from multiple origins. Interestingly, in this experimental setting, LiCl exposure allowed the neutralization of a hMW TCF4 (in particular TCF4E)-dependent transcriptional block and the significant over-expression of specific TCF1 transcriptional targets 54. This report further supports that a similar hMW TCF4 isoform-mediated inhibition of TCF1-induced transcripts may occur also in GBM tumors.

In this study, we describe an unexpected function of HIF-1α as a fundamental tile of the neuronal differentiation process of GBM stem cells. Historically, HIF-1α has been considered as an oncogene in solid tumors, including GBM 7, 12, 55. Similarly, our group previously demonstrated a significant increase of stem cell and chemotherapy resistance traits in GBM cells exposed to low oxygen tensions 11, 30. Nevertheless, HIF-1α possesses a fundamental physiological role of sustaining the process of normal NSC differentiation into neurons in the developing mice brain 15, 56 and eases axonal reconstruction after brain injuries 57. Our results clearly show that a reminiscent neuronal differentiation potential may be engaged also in GBM cells upon Wnt signaling activation and that a proper HIF-1α stabilization is required in order to achieve this task.

In order to validate our results and assign a potential clinical relevance to this HIF-1α/Wnt signaling-dependent control of phenotype, we correlated the co-expression of HIF-1α and TCF1 to the acquisition of neuronal traits in GBM tumors. In this context, a previous study described a regulatory circuit composed of miR-125b/miR-20b and Wnt signaling components able to regulate Mesenchymal (MES) and Proneural (PN) molecular phenotypes of GBM 58, 59. Authors demonstrated that PN GBM tumors are characterized by a regulatory loop, which sustains a strong activation of Wnt signaling, thus counteracting their aggressive behavior. Conversely, MES GBMs seem to be subjected to a consistent attenuation of Wnt signaling, resulting in a much more malignant phenotype 58. In agreement with this model, we found that cells residing in the GBM core or the more peripheral regions display a differential transcriptional enrichment of these peculiar molecular subtypes 59. Indeed, GBM tissues resected from the tumor core were positively enriched for genes of the MES subtype, with more peripheral samples being endowed with PN and N characteristics (Figure S9G-I). Accordingly, our data show that core/peripheral regions of the GBM mass are subjected to a differential activation of Wnt signaling, thus potentially sustaining their reported higher sensitivity to chemotherapy 11. In particular, we hypothesize that a correlation between Wnt signaling activation and specific molecular subtypes might reflect phenotypic heterogeneity at the intra-tumoral level. Indeed, GBM periphery samples resembles PN and N subtype transcriptional features and a concomitant activation of Wnt signaling. Intriguingly, stem cell enriched core biopsies are shown to retain a MES phenotype and an unexpected over-expression of Wnt ligands that, in turn, may exert a persistent stimulation of peripheral cells, due to their intrinsic over-expression of the pathway intracellular effectors (Figure 7F). Interestingly, further supporting our results obtained *in vitro*, although TCF4 levels among different samples resulted highly heterogenous and the antibody used did not allow the discrimination between the different TCF4 isoforms, IHC data suggest a negative correlation of TCF4 with both HIF-1α and βIII-tubulin. Moreover, despite appearing in contrast with our experimental hypothesis, we show that, in general, TCF4 IHC scores are significantly higher in the GBM core than the periphery, however still remaining negatively correlated with HIF-1α when looking at the single sample resolution. In this context, high levels of TCF4 combined to a weaken expression of Wnt signaling components in the core of the GBM mass strongly support the model in which peripheral GBM cells are “enabled” to efficiently transduce the Wnt intracellular signal as we proposed in Figure 7F.

Since we show that GBM tumors characterized by enhanced transcription of at least 7 “Wnt signaling genes” (as described for the GBM periphery) are endowed with a significant better prognosis, our results acquire a strong clinical relevance and suggest that the potential imbalance of the mechanisms sustaining intra-tumoral heterogeneity may dramatically impact on GBM aggressiveness and clinical behavior. Supporting this statement, further analyses conducted by considering a more restricted number of genes (at least 4) endowed with a more substantial up-regulation (z > 2), still show their impact in favoring GBM patient prognosis (data not shown).

Finally, in the last decade, many efforts have been made in order to target GBM stem-like cells. We and others previously suggested pro-differentiating agents as suitable compounds endowed with therapy-sensitizing and anti-tumoral activities against GBM stem cells 31, 32, 60-62. In this context, we believe that a more detailed knowledge of the mechanisms by which HIF-1α and Wnt signaling co-factors can regulate GBM cell phenotype would open the way to a drug-based interference of their molecular competitors/inhibitors, in order to promote GBM cell differentiation and sensitize them to treatments, with the final aim of improving patient survival.

## Supplementary Material

Supplementary figures, and tables S1, S2 and S5.

Supplementary tables S3 and S4.

## Abbreviations

BCBD: β-catenin binding domain
bFGF: basic fibroblast growth factor
BSA: bovine serum albumin
ChIP-seq: chromatin immunoprecipitation sequencing
CSC: cancer stem cell
db-cAMP: (N6,2'-O-dibutyryl)-adenosine-3',5'-mono-phosphate
ddPCR: droplet digital polymerase chain reaction
EGF: epidermal growth factor
FDR: false discovery rate
GBM: glioblastoma
GDNF: glial cell derived neurotrophic factor
GEO: gene expression omnibus
GO: gene ontology
GSEA: gene set enrichment analysis
GSK3: glycogen synthase kinase 3
HIF-1α: hypoxia inducible factor-1α
HMG: high-mobility group
hMW: high molecular weight
IHC: immunohistochemistry
IP: immunoprecipitation
MES: mesenchymal
MRI: magnetic resonance imaging
MTT: 3-(4,5-dimethylthiazol-2-yl)-2,5-diphenyltetrazolium bromide
N: neural
NLS: nuclear localization site
NSCs: neural stem cells
ODD: oxygen dependent domain
PBS: phosphate buffered saline
PN: proneural
PVDF: polyvinylidene difluoride
Q-PCR: quantitative polymerase chain reaction
RLUs: relative light units
RT-PCR: retro-transcription polymerase chain reaction
SAM: significance analysis of microarray
S.E.M.: standard error of the mean
TCGA: the core genome atlas
TMZ: temozolomide
