# Supplementary material for: HIF-1α/Wnt signaling-dependent control of gene transcription regulates neuronal differentiation of glioblastoma stem cells
Source: Theranostics. 2019 Jul 9;9(17):4860–77. doi: 10.7150/thno.35882 (PMC6691379; doi:10.7150/thno.35882)
Supplement: Supplementary file 1 — Supplementary figures, and tables S1, S2 and S5. [file thnov09p4860s1.pdf]

HIF-1α/Wnt signaling-dependent control of gene transcription regulates neuronal differentiation of glioblastoma stem cells.

Daniele Boso *et al.*

SUPPLEMENTARY INFORMATION

FIGURES S1-S8

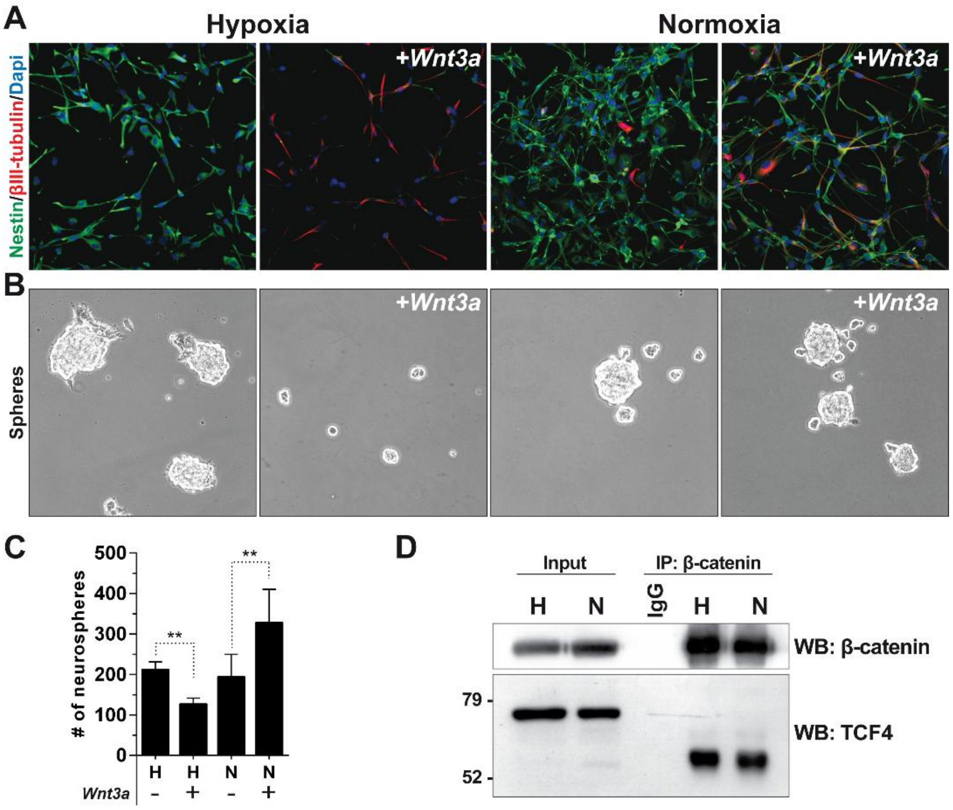

Figure S1. Wnt signaling activation differentially affects the phenotype of GBM cells depending on microenvironmental oxygen. (**A**) Representative immunofluorescence images of H/N GBM cells (HuTuP53) ±Wnt3a (30ng/ml for 4d) stained with Nestin (green) and βIII-tubulin (red) antibodies and counterstained with Dapi (blue). Original magnification 20x. (**B**, **C**) Representative pictures (**B**; HuTuP13) and relative quantification (**C**) of H/N GBM cell neurospheres (n=5) ±Wnt3a (30ng/ml for 4d). (**D**) Representative Western Blot analysis of TCF4 in H/N GBM cells (HuTuP82) in which total protein lysates have been immunoprecipitated for β-catenin. Molecular weights in kDa are reported near WB panels. IP: immunoprecipitation. **p<0.01 by one-way ANOVA. d: days; H: hypoxia; IP: immunoprecipitation; N: normoxia; WB: western blot.

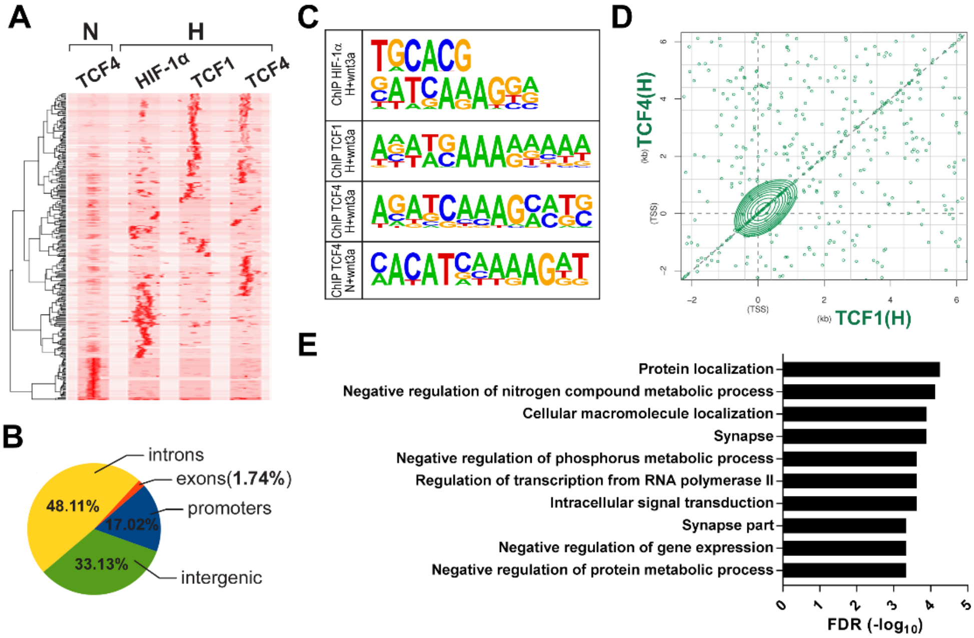

Figure S2. TCF1 and TCF4 bind to specific genomic regions and regulate peculiar gene transcription. (**A**) Heatmap showing the genomic binding within 250bp of peaks selected by pooling the 50 peaks with the highest fold-enrichments and the 50 peaks with the lowest p-values for each of the immunoprecipitated transcription factors (TF) in HuTuP47 cells for a total of 289 unique peaks. (**B**) Peak distribution annotation in relation to gene centered regions (bottom panel). (**C**) Logos of the motifs identified in genomic sequences recognized by HIF-1α, TCF1 and TCF4. (**D**) Scatter plots representing the peak localization around gene TSS of TCF1 and TCF4 under H (green); areas showing higher concentration of peaks are evidenced by contour lines. (**E**) GO analysis of genes bound by TCF1 and TCF4 in H. FDR: false discovery rate; GO: gene ontology; H: hypoxia; N: normoxia; TSS: transcriptional starting site.

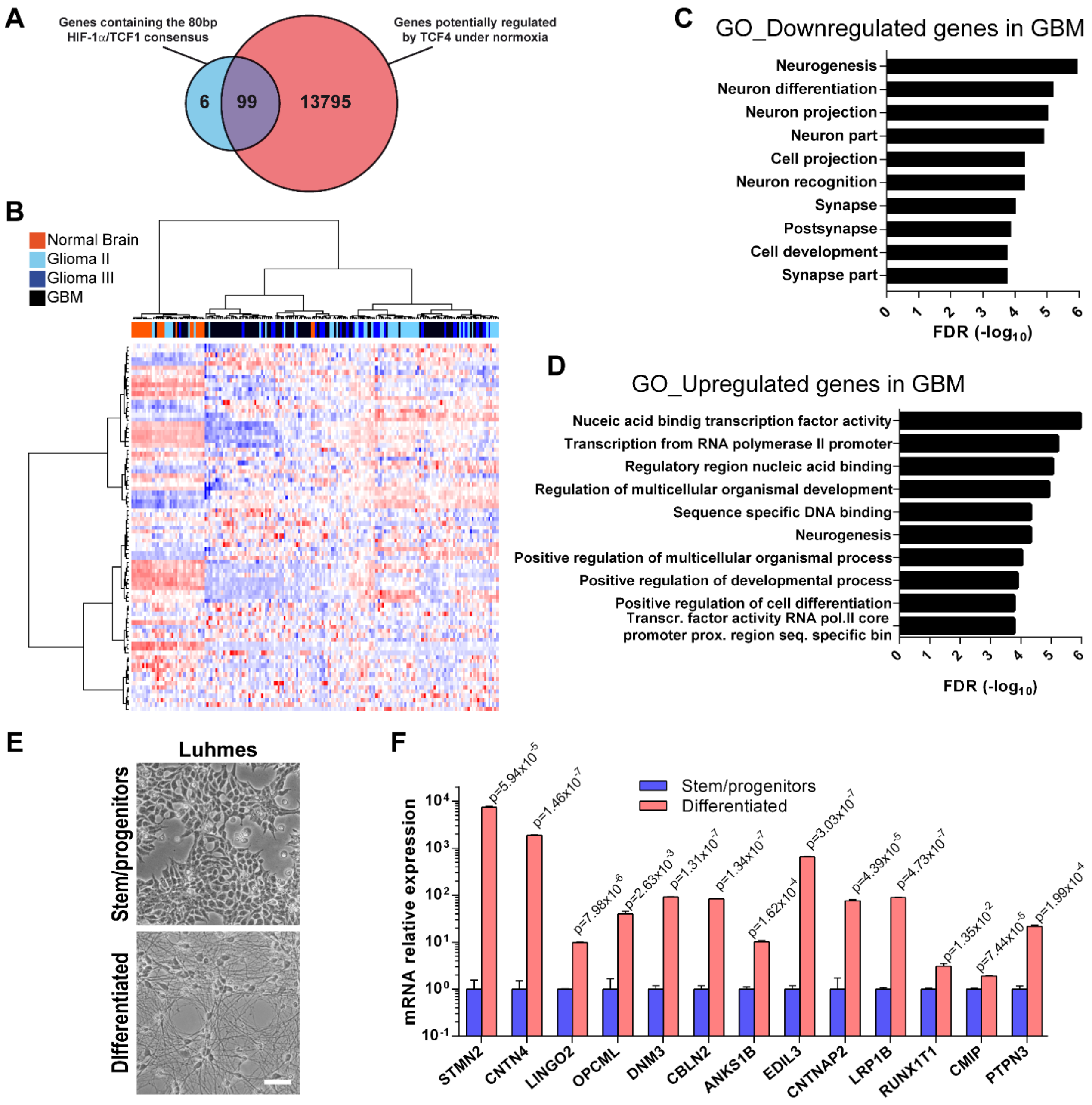

Figure S3. TCF1 and TCF4 regulate the transcription of genes which expression is negatively correlated with glioma grade. (**A**) Venn diagrams representing the intersection between the gene lists retrieved from HIF-1α/TCF1 commonly regulated genes under H (containing a peculiar 80bp regulatory region as described) and genes potentially regulated by TCF4 under N. (**B**) Hierarchical clustering analysis of the gene signature generated from the intersection of genes in (**A**) (85 genes) applied to the GSE4290 dataset. (**C**, **D)** GO analysis of up-regulated (**C**, n=35) and down-regulated genes (**D**, n=50) between GBM and normal brain samples from GSE4290 dataset. (**E**) Representative brightfield images displaying the morphology of Luhmes cells when cultured in stem cell medium (up) or after exposure to 10 days of neuronal differentiation induction cocktail (bottom). Original magnification 10x; bar=25µm. (**F**) Bar graph showing the expression of top down-regulated genes from Fig. 2E (13/20) in Luhmes cells when cultured in stem cell medium (blue) or upon 10 days of differentiation (red). FDR: false discovery rate; GO: gene ontology; H: hypoxia; N: normoxia.

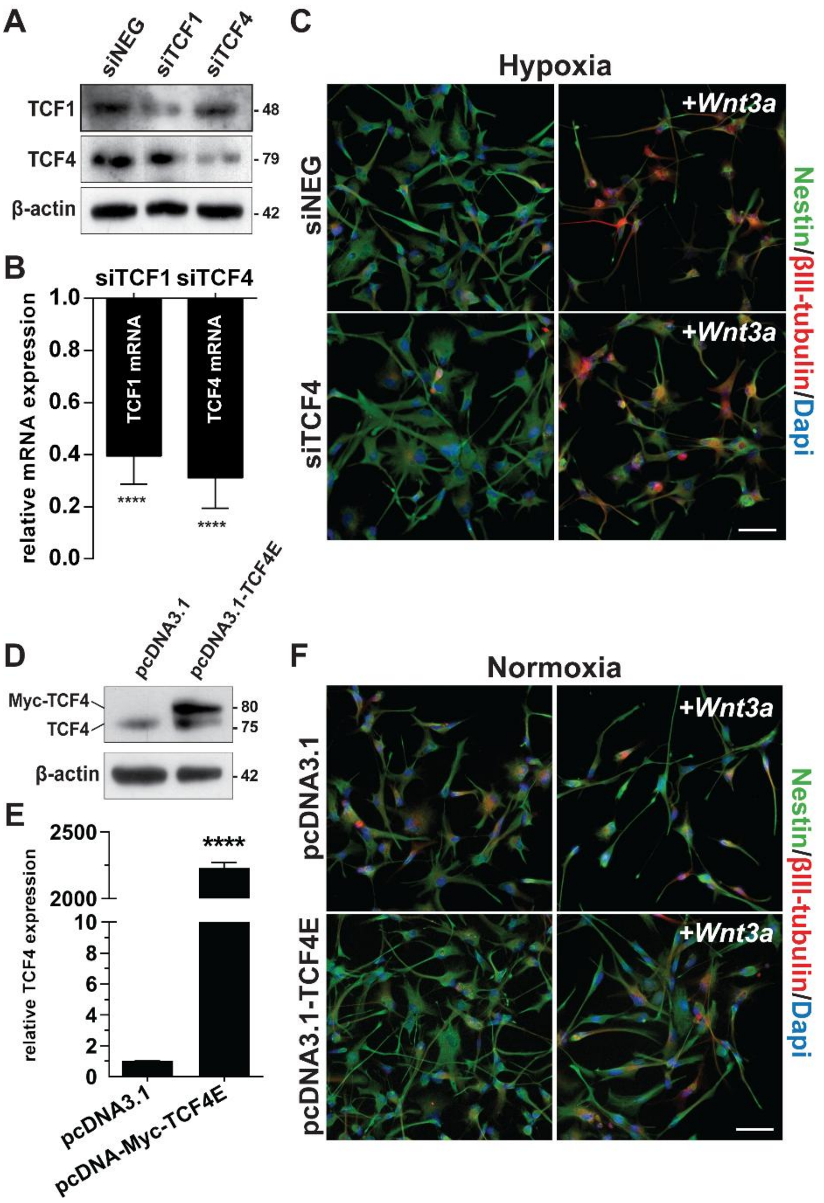

Figure S4. TCF4 levels are inversely correlated to neuronal differentiation in GBM cells. (**A**, **B**) Representative analysis of TCF1 and TCF4 protein (**A**) and mRNA (**B**) expression upon their silencing with specific siRNAs by WB (HuTuP53) and RT-PCR (n=3), respectively. siNEG was used as negative control of knockdown and for normalization in RT-PCR. β-actin was used as loading control. (**C**) Representative immunofluorescence images of hypoxic siNEG/siTCF4-GBM cells (HuTuP13) treated with Wnt3a (30ng/ml for 4d) and stained with Nestin (green) and βIII-tubulin (red) antibodies. (**D**, **E**) Representative analysis of TCF4E protein (**D**) and mRNA (**E**) expression upon pcDNA3.1-TCF4E transfection by WB (HuTuP53) and RT-PCR (n=3), respectively. Empty pcDNA3.1 construct was used as negative control of over-expression and for normalization in RT-PCR. β-actin was used as loading control. (**F**) Representative immunofluorescence images of normoxic TCF4E-GBM cells (HuTuP53) treated with Wnt3a (30ng/ml for 4d) and stained with Nestin (green) and βIII-tubulin (red) antibodies. In WB molecular weights in kDa are reported near panels. In (**C**, **F**) original magnification 20x; bar=50μm. In all images cell nuclei have been counterstained with Dapi (blue). ****$p<0.0001$ by paired *t*-test. d: days; WB: western blot.

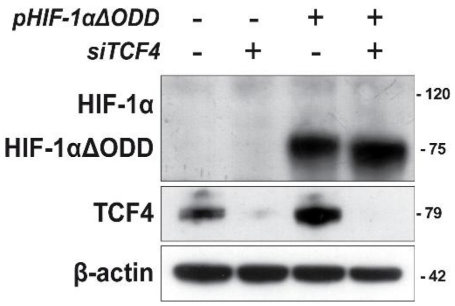

Figure S5. Concomitant transfection of a constitutive activated form of HIF-1α and TCF4 siRNA. Representative Western Blot analysis of HIF-1α and TCF4 in H/N GBM cells (HuTuP53) in which siTCF4 or pcDNA3.1-HIF-1αΔODD or both have been transiently transfected. β-actin was used as loading control. Molecular weights in kDa are reported near WB panels. H: hypoxia; N: normoxia.

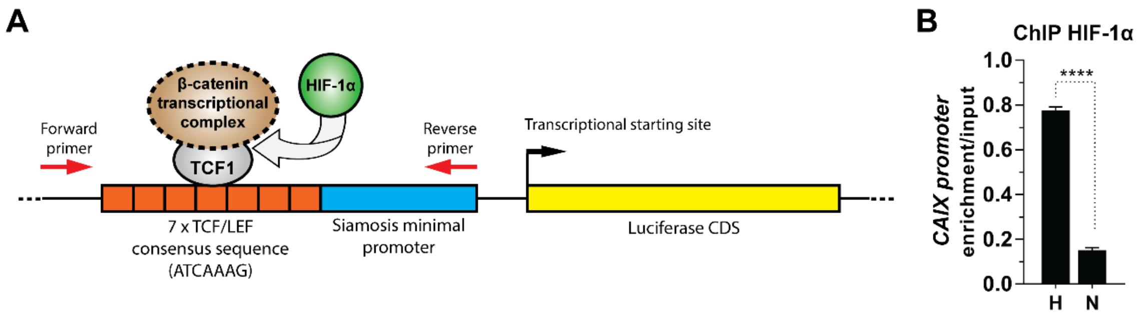

Figure S6. TCF1 knockdown impairs HIF-1α localization onto TCF/LEF consensus sequences. (**A**) Graphic model of the 7xTCF/LEF binding site-based BAT-lux reporter construct used for analyzing the binding of HIF-1α upon TCF/LEF consensus sequences. Primers used for amplifying the HIF-1α-cross-linked plasmid sequences flanking the Wnt-responsive promotorial region for ddPCR experiments are highlighted as red arrows. (**B**) Bar graph reporting the relative enrichment of *CAIX* promoter sequences co-immunoprecipitated with HIF-1α under H or N, measured by ddPCR amplification (n=3). ****p<0.0001 by paired *t*-test. ddPCR: droplet digital PCR; H: hypoxia; N: normoxia.

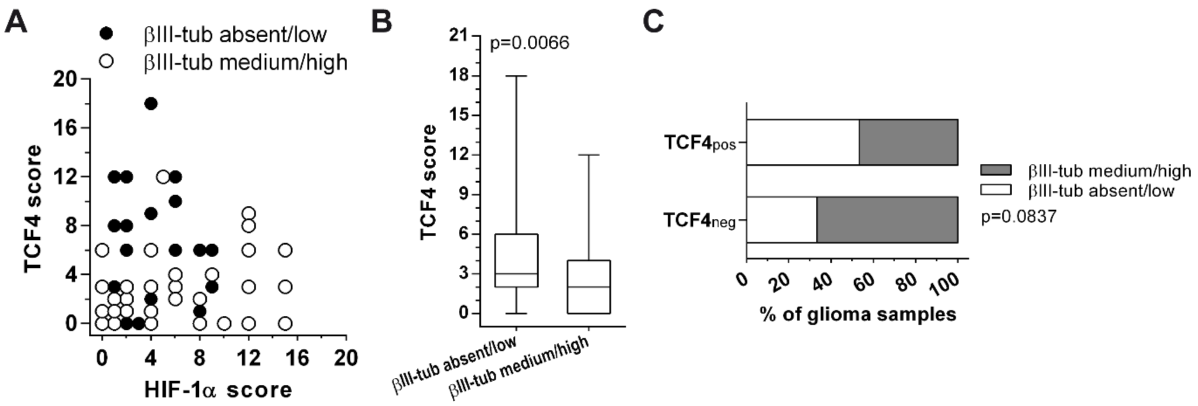

Figure S7. TCF4 is negatively correlated with neuronal differentiation. (**A**) Correlation graph suggesting the existence of a negative correlation between TCF4 (score), HIF-1α (score). (**B**) Box plot reporting the quantification of TCF4 score in our cohort of glioma samples and showing a significant negative correlation with neuronal differentiation (p value calculated by Mann-Whitney). (**C**) Bar graph showing the enrichment (by chi-square analysis) of βIII-tubulin expressing (medium/high intensity) biopsies in $TCF4_{neg}$ and $TCF4_{pos}$ glioma samples. neg: negative; pos: positive.

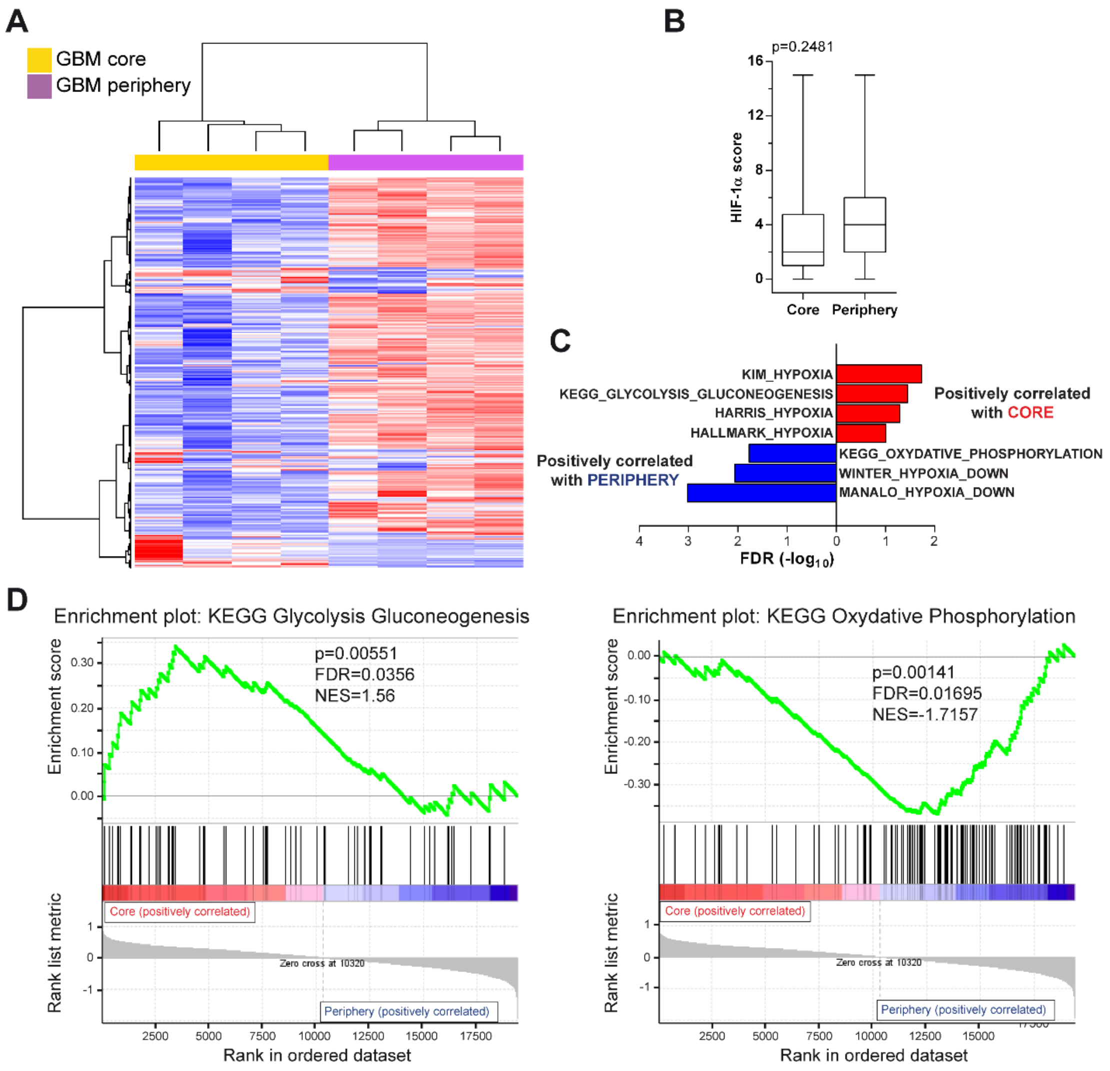

Figure S8. GBM samples from different regions of the mass show a differential transcriptional enrichment of a HIF-1α-dependent gene signature. (**A**) Heatmap and hierarchical clustering analysis of differentially expressed genes ($p<0.05$, FDR q value<0.05) between GBM samples (n=4) derived from the Core and the Periphery of the mass. (**B**) Box plot reporting the quantification of HIF-1α score in GBM Core and Periphery (p value calculated by Mann-Whitney). (**C**) Bar graph representation of GSEA for gene sets either enriched in the GBM core or periphery. (**D**) Representative GSEA of differentially expressed genes between Core and Periphery showing significant positive enrichment for KEGG Glycolysis Gluconeogenesis gene set in GBM core and positive enrichment for KEGG Oxydative phosphorylation gene set in GBM periphery. FDR: false discovery rate; GSEA: gene set enrichment analysis; NES: normalized enriched score.

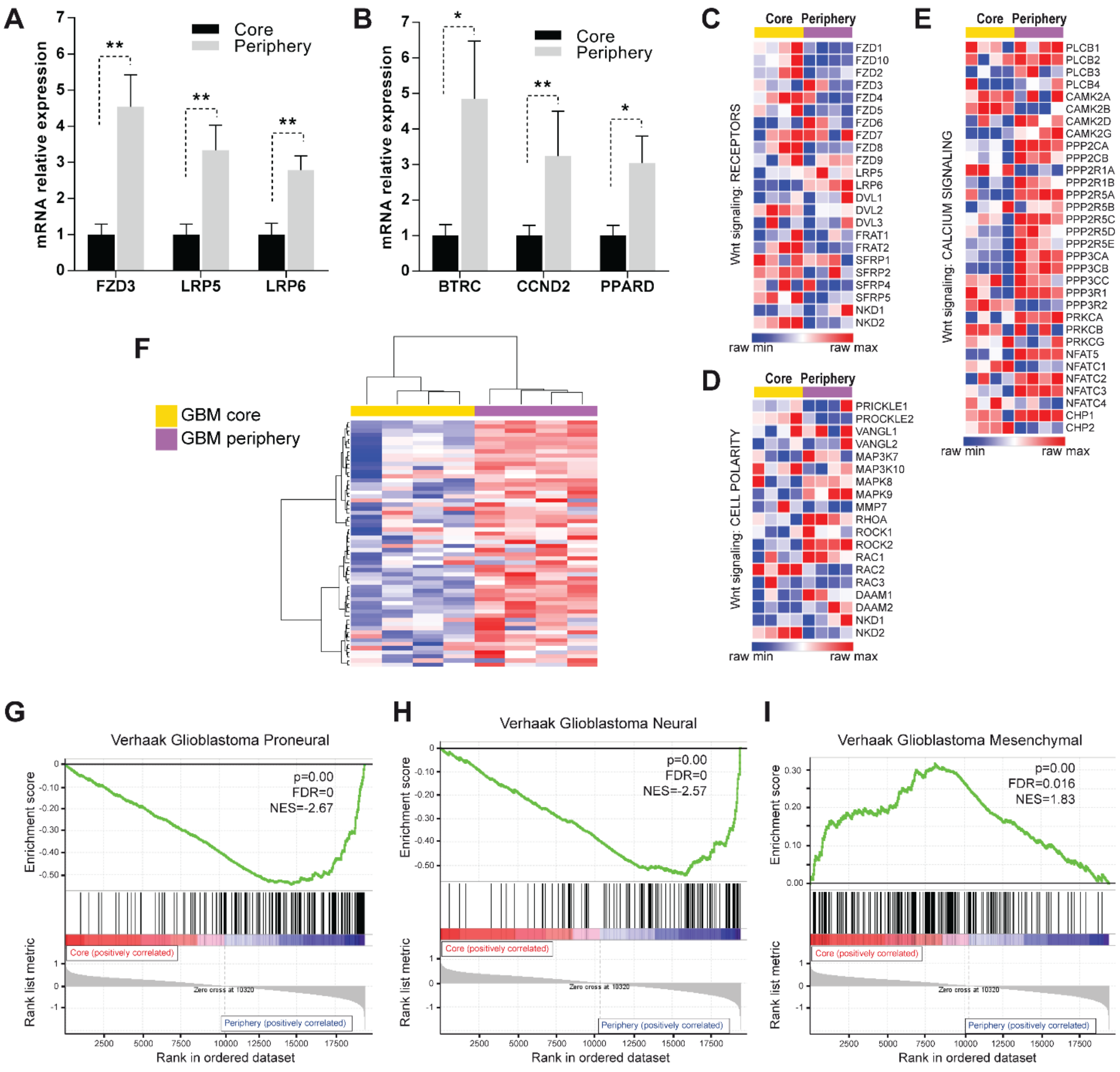

Figure S9. GBM samples from different regions of the mass show a differential transcriptional enrichment of Wnt signaling components and GBM molecular subtype features. (**A**, **B**) Bar graphs reporting a differential mRNA relative expression of Wnt signaling receptors (**A**; *FZD3*, *LRP5* and *LRP6*) and intracellular components (**B**; *BTRC*, *CCND2*, *PPARD*) between GBM samples derived from the Core and the Periphery of the mass (n=10). (**C-E**) Level plots reporting the differential expression of Wnt signaling receptors (**C**), Wnt signaling-dependent calcium signaling components (**D**) or Wnt signaling-dependent cell polarity regulators (**E**) between GBM Core and Periphery. (**F**) Heatmap and hierarchical clustering analysis of 60/84 genes previously demonstrated to share a peculiar transcriptional control exerted by HIF-1α/TCF1 in hypoxia and TCF4 in normoxia (Fig. 2C-E) which show a significant over-expression in GBM periphery. (**G-I**) GSEA of differentially expressed genes between GBM Core and Periphery showing that Core biopsies are negatively enriched for Proneural (**G**) and Neural (**H**) transcriptional features, but positively correlated to a Mesenchymal GBM phenotype (**I**). FDR: false discovery rate; GSEA: gene set enrichment analysis; NES: normalized enrichment score.

TABLES S1, S2 and S5

Table S1. Clinical features of glioblastoma patients from which primary cell cultures used in this study have been derived.

Patient ID	Diagnosis	WHO grade	Gender	Age (y)
**HuTuP01**	GBM	IV	Male	64
**HuTuP10**	GBM	IV	Female	75
**HuTuP13**	GBM	IV	Male	67
**HuTuP15**	GBM	IV	Female	76
**HuTuP36**	GBM	IV	Female	49
**HuTuP43**	GBM	IV	Male	59
**HuTuP47**	GBM	IV	Female	81
**HuTuP53**	GBM	IV	Male	70
**HutuP82**	GBM	IV	Male	50
**HuTuP83**	GBM	IV	Male	55
**HuTuP108**	GBM	IV	Male	62
**HuTuP174**	GBM	IV	Male	69
**HuTuP175**	GBM	IV	Female	57
**HuTuP187**	GBM	IV	Male	56
**HuTuP197**	GBM	IV	Male	48

Table S2. Clinical features of glioblastoma patients from which total RNA has been extracted from core and peripheral GBM tissues according to a multiple sampling procedure as described in the Material and methods section.

Patient ID	Diagnosis	WHO grade	Gender	Age (y)	RT-PCR	GEP
**HuTuP64**	Glioblastoma	IV	Male	60	•	•
**HutuP67**	Glioblastoma	IV	Male	48	•	
**HutuP70**	Glioblastoma	IV	Male	40	•	
**HuTuP95**	Glioblastoma	IV	Male	66	•	
**HuTuP107**	Glioblastoma	IV	Male	65	•	•
**HuTuP109**	Glioblastoma	IV	Male	58	•	•
**HuTuP119**	Glioblastoma	IV	Male	54	•	•
**HutuP120**	Glioblastoma	IV	Male	54	•	
**HutuP144**	Glioblastoma	IV	Female	46	•	
**HutuP169**	Glioblastoma	IV	Female	61	•	
**HutuP185**	Glioblastoma	IV	Male	59	•	

Table S5. Sequence of primers used in this study.

GENE	FORWARD PRIMER (5'-3')	REVERSE PRIMER (5'-3')
BAT-lux plasmid	CGCGGGAATTCGATTAAGGAC	AACAGGGGACAAAGGGTGTG
TCF7	CCTAGCAAGGAGGAGCGAGA	CCGGTTGGCAAACCAGTTGTAG
TCF7L2	TTTAAGGGGCCACCGTATCC	TGCCGGACTGAAAATGGAG
CHRM3	GCCGGGATCATCATGACCGT	TGCATCGGAGGGGCTGTGTAT
CMIP	GGGGTCTCGCACAGGTTCAG	GGGGTCTCGCACAGGTTCAG
LRP1B	AGATGCTGTGGCCAAACGGT	TGCGACAGTCCGAAAGGGTG
LRP5	AACGGCAGGACGTGTAAGGC	AGCCCTCTAGCGGGTCGTAG
LRP6	CGCCGGTGAGAGAAGAGAACG	AACCAATCGCAAGTCCCGTCT
FZD3	CCAACAGACAGCAGCTTTGGC	TAAGCCCGCTGACACAGCCT
BTRC	CCTCTGATGGCATGCTGTGGA	TGGGAGGAGCATTCCCGTCA
CCND2	GTGGGAGCAGCCATCTGTGG	GAGCACCGCCTCAATCTGCT
PPARD	GGCCCTATTCATTGCGGCCA	CTGGGCATCAGGGTGGTTGG
CA9	CAGTTGCTGTCTCGCTTGGA	TCAGAGGGCAGGAGTGCA
GUSB	GAAAATACGTGGTTGGAGAGCTCATT	CCGAGTGAAGATCCCCTTTTTA
STMN2	TTCAGCAAGATGGCGGAGGA	ATGCCTCTCATTGCTTCTCTCCTT
CNTN4	ACCACTTTGAAAGAGTTGGAGGGC	ATGGGGCTGTGGTTGTCAGG
LINGO2	AGCAGAGCACCGAAAGTGGC	ACGAACCTTGAAAGCTGCATTCTG
OPCML	TGTTCCTTGTACCCACAGGAGTG	TGACCAGGATGATCACACGAGGG
DNM3	TCCTGACAAATCTGTAGGGAACA	GAGGTTGCGAATGGTCTCCA
CBLN2	CGAGATGAGCAACCGCACCA	GGTCTCCTGCAAAGGCCGAG
ANKS1B	AGGGGGACACTCCTCCACAC	CTGATGGTTCCTGCCCGGTAT
EDIL3	AGCTCGGCTGGACAAGCAAG	TGCCAGTCACTTTGGTTGGAACA
CNTNAP2	GTCAACATCACCCGCCACGA	GCCTTGAGAGGGGCGATCTG
LRP1B	AGATGCTGTGGCCAAACGGT	TGCGACAGTCCGAAAGGGTG
RUNX1T1	CCGGAGTGTCAGCTCTCCATC	ACATCCACAGGTGAGTCTGGC
CMIP	CCCTCCAAGTCCACAGACGC	GGGGTCTCGCACAGGTTCAG
PTPN3	CGGTTACGTGCGTTGGGTG	CCCAGGTGGTTGTGCACCATA
PAK1	GCTGCTACAGGTGAGAAAACTGAGG	AGGCTTGGCACAATGAGGCT
